# Efficient and iterative retron-mediated in vivo recombineering in Escherichia coli

**DOI:** 10.1093/synbio/ysac007

**Published:** 2022-05-03

**Authors:** Adam J Ellington, Christopher R Reisch

**Affiliations:** Department of Microbiology and Cell Science, Institute of Food and Agricultural Sciences, University of Florida, Gainesville, FL, USA; Department of Microbiology and Cell Science, Institute of Food and Agricultural Sciences, University of Florida, Gainesville, FL, USA

**Keywords:** genome editing, retron, recombineering, Cas9, MutL_E32K_

## Abstract

Recombineering is an important tool in gene editing, enabling fast, precise and highly specific *in vivo* modification of microbial genomes. Oligonucleotide-mediated recombineering via the *in vivo* production of single-stranded DNA can overcome the limitations of traditional recombineering methods that rely on the exogenous delivery of editing templates. By modifying a previously reported plasmid-based system for fully *in vivo* single-stranded DNA recombineering, we demonstrate iterative editing of independent loci by utilizing a temperature-sensitive origin of replication for easy curing of the editing plasmid from recombinant cells. Optimization of the promoters driving the expression of the system’s functional components, combined with targeted counterselection against unedited cells with Cas9 nuclease, enabled editing efficiencies of 90–100%. The addition of a dominant-negative *mutL* allele to the system allowed single-nucleotide edits that were otherwise unachievable due to mismatch repair. Finally, we tested alternative recombinases and found that efficiency significantly increased for some targets. Requiring only a single cloning step for retargeting, our system provides an easy-to-use method for rapid, efficient construction of desired mutants.

Graphical Abstract

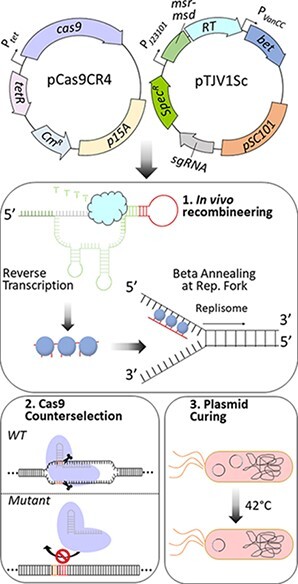

## 1. Introduction

Genome editing technologies have proven to be invaluable molecular tools, enabling rapid advances in functional genomics, metabolic engineering, therapeutics development, bioremediation and more (1–4). Traditional methods for genome editing use allelic exchange via the RecA-dependent process of homologous recombination (HR). In HR, circular or linear DNA substrates, encoding the desired modification flanked by targeted regions of homology, are introduced into recombination-proficient (Δ*recBC*Δ*sbcBC* or Δ*recD*) cells (5, 6). Endogenous recombination proteins facilitate crossover between the editing template and the target locus, incorporating the desired mutations into the host chromosome. While this process is useful for targeted mutagenesis, it is sometimes inefficient and always laborious, requiring numerous cloning steps to incorporate sizable regions of homology (0.5–5 kb) and extensive screening to identify recombinant cells (7).

The development of *in vivo* recombination-mediated engineering (recombineering), which relies on phage-derived proteins to facilitate DNA integration, has greatly improved the speed and efficiency with which targeted mutations can be made. For example, the λ-phage-derived Red system in *Escherichia coli* yields more than a 100-fold increase in recombinant cells over traditional HR through a process independent of RecA (8, 9). Recombineering reduces editing template design constraints by enabling efficient recombination with homology arms of <50 bp on double-stranded DNA (10). In addition, short single-stranded DNA (ssDNA) oligonucleotides (oligos) can be used as suitable recombineering substrates. In fact, ssDNA oligos have produced recombinant cells with just one of the three λ-Red proteins, the ssDNA-annealing protein Beta, further simplifying the requirements for successful recombineering (9). Furthermore, the Clustered Regularly Interspaced Short Palindromic Repeats (CRISPR)–Cas9 bacterial immune system has been adapted as a programmable counterselection tool to selectively target and kill unedited cells after recombineering. This technique enhances the apparent efficiency by increasing the mutant to wild-type ratio of the population, allowing highly efficient scarless genome editing (11–14). Additional efforts for the improvement of λ-Red editing efficiency have used engineered mutator strains, where the overexpression of HR-involved enzymes, the deletion of host exonucleases and/or disabling the mismatch repair system significantly improve recombination efficiency but can unintentionally lead to the accumulation of off-target mutations (15–17).

**Figure 1.**
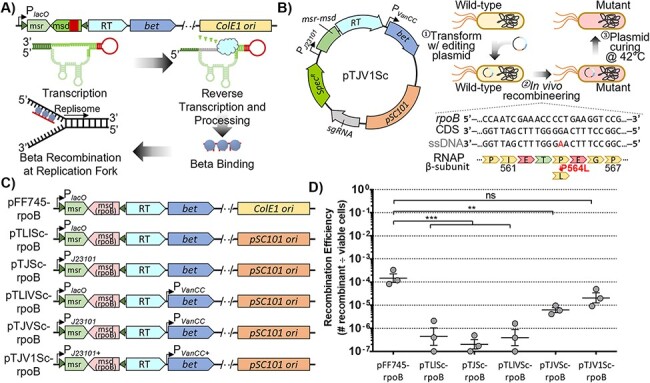
Optimization of the origin of replication and promoter elements enables an efficient and curable system for *in vivo* recombineering . (A) *In vivo* retron-generated ssDNA serves as an editing substrate for beta recombination at the lagging strand of the replication fork. (B) Rifampicin resistance conferred by the P564L mutation of the *rpoB* gene was used to measure the recombination efficiency. The pSC101 origin of replication allows for curing of the editing plasmid by growth at 42°C after desired mutation(s) are made. (C) Modifications made to promoters and origin of replication for enhanced recombination efficiency and curability. (D) Recombination efficiency of each editing plasmid reported as the average of three independent replicates. Error bars represent SEM, and statistical significance relative to pFF745–rpoB is denoted by asterisks (ns = not significant; **P < 0.01; ***P < 0.001; one-way analysis of variance and Tukey’s test of log-transformed values).

Farzadfard and Lu (18) described a novel approach for recombineering, termed Synthetic Cellular Recorders Integrating Biological Events (SCRIBE), where the ssDNA is generated *in vivo* and then incorporated into the chromosome. The *in vivo* ssDNA is produced by the Ec86 retron of *E. coli* BL21, composed of the msr and msd RNA elements and the reverse transcriptase (RT). Upon transcription of the *msr–msd* sequence, inverted repeats flanking the msr–msd RNA form a secondary structure specifically recognized by the RT and the msdRNA is reverse transcribed to ssDNA. Simultaneous expression of *bet* on the SCRIBE plasmid allows for efficient recombination of the newly synthesized ssDNA into the lagging strand of the targeted locus during DNA replication (Figure 1A). By placing control of this system under an inducible promoter, the cells record their exposure to the inducer as the fraction of cells with the *msd* encoded mutation. By simply changing the *msd* sequence, SCRIBE can be easily retargeted to modify any desired genomic site. The authors report a maximum efficiency for SCRIBE of 10^–4^ recombinants per generation (18). While sufficient for making mutations conferring easily selectable phenotypes, this rate of recombination is not robust enough to obviate the need for extensive screening when making nonselectable edits (19).

Farzadfard *et al.* (20) improve upon their original SCRIBE design to achieve highly efficient ssDNA recombineering via transient knockdown of host exonuclease expression through CRISPR interference (CRISPRi), reporting nearly 100% recombination efficiency for one of their chosen targets. Additionally, the authors demonstrate that SCRIBE enables the incorporation of multiple mutations at distinct loci (18, 20). However, with no easy method for plasmid curing, doing so requires that each editing plasmid contain a unique selection marker for plasmid maintenance, a strategy limited by the number of orthogonal selection markers and compatible plasmid origins available.

In this work, we sought to enable iterative genome editing by moving the functional components to easily curable plasmids while maintaining the SCRIBE system’s high recombination efficiency. We hypothesized that combining an improved SCRIBE system with Cas9 counterselection against wild-type cells would provide an easy-to-use genome editing platform that precluded the need to electroporate ssDNA for recombineering. In addition, the system was designed so that the editing plasmid can be retargeted through a single round of PCR amplification and cloning to reduce construction times and enable the rapid generation of multilocus mutants.

## 2. Materials and methods

### 2.1 Strains, plasmids and culture conditions

Bacterial strains used in this work are listed in Supplementary Table S1. Plasmid construction methods and primers are described in Supplementary Table S2 (along with Addgene #s for available plasmids) and the Primers_GeneFrags.fa file, respectively. The pFF745 plasmid was a gift from Timothy Lu (Addgene #61450). Cloning steps for retargeting the editing plasmid are illustrated in Supplementary Figure S2. Briefly, primers with overhangs encoding the new target sequence were used to PCR amplify the vector using Q5 High-Fidelity 2x Master Mix (New England Biolabs, NEB), followed by DpnI digestion to remove template DNA, agarose gel electrophoresis to verify amplicon size and column-based DNA cleanup. DNA fragments were then assembled using the 2x NEBuilder HiFi DNA Assembly Master Mix (NEB) with the following modifications: 1 μl Master Mix + 0.5 μl each DNA fragment, incubated at 50°C for 15 min. Chemically competent *E. coli* NEB5α cells were used for cloning. Antibiotics were used at the following concentrations unless otherwise noted: spectinomycin (50 μg/ml), kanamycin (50 μg/ml), chloramphenicol (40 μg/ml), rifampicin (20 μg/ml), tetracycline (20 μg/ml) and carbenicillin (100 μg/ml). The *tetA* gene was PCR amplified from pUC18-mini-Tn7T-Gm-TetAR using primers 2490 and 2491 and integrated into *E. coli* MG1655 using λ-Red recombination with pKD46 (21), creating the MG1655 *yhiS::tetAR* strain.

### 2.2 Recombineering assays and recombination efficiency determination

Since the number of recombinant cells should increase with time, all experiments were performed in freshly transformed cells. Approximately 100 ng of editing plasmid was transformed into competent *E. coli* cells via either heat shock at 42°C or electroporation in a 0.1-cm gap cuvette with a single 1.8-kV pulse. Cells were recovered in 1 ml SOB at 30°C for 2 h and then outgrown overnight at 30°C in 5 ml Luria Bertani (LB) broth + appropriate antibiotic(s) to select transformants. The total number of viable cells was determined by spotting 10 µl 10-fold serially diluted overnight cultures on LB plates + appropriate antibiotic(s). For the *rpoB* and *tetR* assays, 10 μL of 10-fold serially diluted overnight cultures were spotted on LB plates with rifampicin or tetracycline to determine the number of recombinant cells. For the *ackA* assays, 1 ml of cells from overnight cultures were pelleted, washed and resuspended with sterile phosphate buffered saline after which washed cells were spotted on M9 chloroacetate plates (M9 minimal media + 10 mM sodium chloroacetate + 2% glycerol + 0.1% SOB) to determine the number of *ackA* mutant cells. Recombination efficiency was determined by dividing the number of recombinant cells by the total number of viable transformants. Efficiencies reported are the mean and standard error for three independent replicates. For the *rpoB* experiments, spontaneous rifampicin resistance was assessed for the wild-type strain as described in Section 2.6 below and the spontaneous mutation rate was subtracted from all samples plated on rifampicin. All statistical analyses were performed using the GraphPad Prism 5 program.

### 2.3 Promoter optimization

The P*_lacO_* promoter of the Ec86 retron cassette in pTLlSc-rpoB was replaced with P*_J23101_*, and P*_VanCC_* was inserted upstream of *bet* as described in Supplementary Table S2. Expression of the Ec86 retron cassette and beta recombinase by P*_J23101_* and P*_VanCC_*, respectively, was further optimized by PCR amplifying pTJVSc-rpoB with primers 2326 & 2358 and 2325 & 2359, followed by assembly of the two resulting amplicons using the 2x NEBuilder HiFi DNA Assembly Master Mix (NEB). The assembled pTJVSc-rpoB promoter library was transformed into *E. coli* NEB5α cells, and recombination efficiency was determined. In addition, the remainder of the pTJVSc-rpoB culture was diluted 1:100 into 5 ml LB broth + rifampicin and grown overnight again at 30°C. The following morning, the plasmid library was extracted, retransformed into *E. coli* NEB5α cells, and the process was repeated three more times. After the fourth round of selection, the pTJVSc-rpoB plasmid was extracted from four randomly chosen transformants and the P*_J23101_* and P*_VanCC_* promoters were sequenced with primers 746 and 2252, respectively, to assess the convergence of the population on a single optimal promoter sequence, yielding pTJV1Sc-rpoB as indicated in Supplementary Table S2.

### 2.4 Cas9 counterselection

For pCas9CR4 for CRISPR/Cas9 directed counterselection against unedited wild-type cells, transformants with editing plasmid were outgrown overnight. The following morning cultures were diluted 1:10 into LB + appropriate antibiotics with or without anhydrotetracycline (aTc; 0.1 μg/ml) to induce Cas9 expression and then grown overnight at 30°C. Serial dilutions were spotted onto LB + rifampicin for the *rpoB* assays, LB + tetracycline for the *tetA* reversion assays, or M9 chloroacetate for the *ackA* assays, then incubated overnight at 37°C. The efficiency of Cas9 counterselection was determined by dividing the number of colonies from the aTc-induced cultures that grew with selection by the total number of viable cells plated on a nonselective plate. Results are reported as the mean with standard error for three independent replicates.

### 2.5 Efficiency improvement with negative mutator alleles

The *mutL* gene was cloned into pCas9CR4 as described in Supplementary Table S2. The *cymR* repressor and P*_cymRC_* promoter (22) were inserted upstream of *mutL* to allow for inducible expression by the addition of cumate, yielding pCas9CyMutL. *Escherichia coli* MG1655 cells harboring pCas9CyMutL were then transformed with pTJV1Sc-rpoB1 or pTJV1Sc-tetA and recovered in 1 ml SOB at 30°C for 2 h, after which cells were transferred to 5 ml LB + chloramphenicol + spectinomycin with or without cumate (100 μM) and outgrown overnight at 30°C. Cultures were diluted 1:10 into LB + chloramphenicol + spectinomycin + cumate with or without aTc (0.1 μg/ml) and grown overnight at 30°C. Recombination efficiency and efficiency of Cas9 counterselection were determined the following day, and the results are reported as the mean with standard error for three independent replicates.

### 2.6 Off-target mutation frequency analysis

*Escherichia coli* MG1655 was separately transformed with pTV1β-rpoB and pCas9CyMutL and plated on LB + spectinomycin and LB + chloramphenicol, respectively, for the selection of transformants. Single colonies from each transformation were used to inoculate 5 ml LB broth + appropriate antibiotic in triplicate and grown overnight at 30°C, with pCas9CyMutL grown with or without cumate (100 μM). Cultures were then diluted 1:10 into the same media and grown for an additional overnight at 30°C, after which 10 μL of 10-fold serial dilutions of each were spotted onto LB only and LB + rifampicin to assess the spontaneous mutation frequency.

For genome sequencing, a single colony from each transformation and wild-type MG1655 were grown in LB broth + appropriate antibiotic overnight at 30°C, with pCas9CyMutL grown with and without cumate (100 μM) for induction of *mutL* expression. Cultures were then diluted 1:100, grown overnight and repeated for a total of two setbacks. Genomic DNA was extracted after the first setback for wild-type and after the second setback for the other samples using a cetyltrimethyl ammonium bromide (CTAB)/phenol-chloroform extraction protocol (23).

Samples were prepared for whole-genome sequencing using the NEBNext Ultra II FS DNA Library Prep Kit for Illumina (NEB). Sequencing was performed using the NovaSeq6000 sequencing platform (Novogene Co. Sacramento, CA). Sequencing data were quality filtered and adapters were trimmed using the Trim Galore script (24). Mutations were identified using the Breseq mutational analysis pipeline (25) set to polymorphism mode with the default parameters and a minimum coverage cutoff of 20× reads. The *E. coli* MG1655 reference genome (National Center for Biotechnology Information (NCBI) accession: NC_000913) was used as the reference sequence. The wild-type MG1655 parent strain was used as a control to assess differences between the reference genome and our lab strain and sequence variations identified were subtracted from those found in our experimental samples.

## 3. Results

### 3.1 Improvement of recombineering with reverse-transcribed ssDNA

To enable an efficient and iterative system for genome editing with ssDNA reverse-transcribed *in vivo*, we moved the SCRIBE system’s functional components to the pKDsgRNA plasmid that has the temperature-sensitive variant of the pSC101 origin to allow for easy curing with growth at 37–42°C (21, 26, 27). This plasmid, pTLlSc, possesses the *msr-msd* coding sequence and Ec86 RT for the synthesis of ssDNA, the *bet* gene to facilitate incorporation of the ssDNA and the *S. pyogenes* single-guide RNA (sgRNA) to enable Cas9 targeting. The judicious design of this plasmid enables easy one-step cloning to retarget both the *msd* and sgRNA (Supplementary Figure S2). To assess the recombination efficiency using an easily selectable mutation, the *msr-msd* was retargeted to introduce three consecutive nucleotide substitutions and create the P564L point mutation of *rpoB* that confers rifampicin resistance (Figure 1B). Here, we define recombination efficiency as the number of cells obtained on selective media divided by the total number of viable cells. First, we compared the efficiency of pFF745, described by Farzadfard *et al.*, which possesses a high-copy pUC origin targeted to *rpoB*, with our temperature-sensitive plasmid pTLlSc-rpoB. As expected, recombination efficiency was about 100-fold lower with the temperature-sensitive plasmid, presumably because of decreased expression of the retron elements and *bet* compared to the high-copy pUC origin on pFF745 (Figure 1D). We sought to improve this efficiency by increasing the transcription of the *msr-msd*, RT and *bet* through promoter replacements. The strong constitutive promoter P*_J23101_* (28) replaced the P*_lacO_* promoter driving expression of the msr-msd RNA and RT. The promoter of *bet* was changed to P*_VanCC_* (22) because it is both strong and nonhomologous to P*_J23101_*. Replacing these promoters individually did not increase recombination efficiency, but when combined, efficiency increased nearly 10-fold (Figure 1D).

To further increase recombination efficiency, we used a selection strategy where a library of variant plasmids was created by cloning degeneracies into both the P*_J23101_* and P*_VanCC_* promoters. We hypothesized that variants with increased efficiency would overtake the population after repeated rounds of outgrowth, selection, plasmid extraction and re-transformation (Supplementary Figure S1A). After four passages, the efficiency of the evolved pool was slightly higher than the original construct (Supplementary Figure S1B), and sequencing of the promoter from randomly chosen transformants revealed a convergence of the pool toward a single promoter sequence that had a recombination efficiency of nearly 10-fold greater than the parent plasmid (Supplementary Figure S1C–D). Moreover, the efficiency of the improved construct, pTJV1Sc-rpoB, was similar to the original SCRIBE plasmid, pFF745–rpoB (Figure 1D).

### 3.2 CRISPR/Cas9 counterselection against wild-type cells

**Figure 2.**
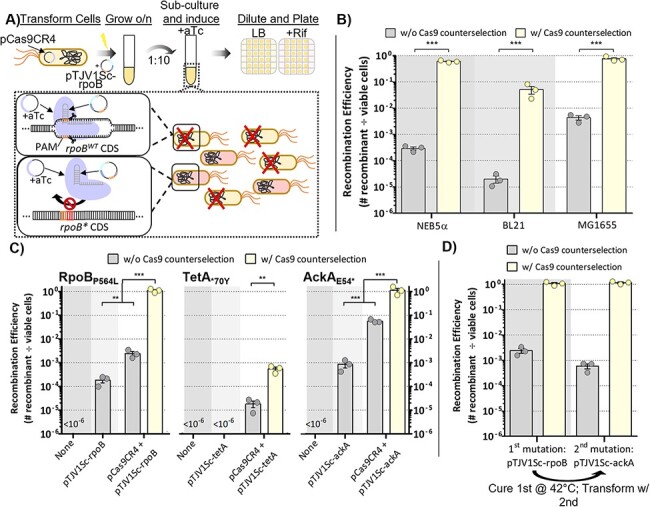
Targeted counterselection against unedited cells by inducible cas9 expression. (A) After Beta-recombination, cells are sub-cultured 1:10 into media with aTc to induce *cas9* expression from pCasCR4. Constitutive expression of an sgRNA from pTJV1Sc-rpoB targets *rpoB*^WT^ alleles for DNA DSB by Cas9 cleavage, thereby eliminating nonmutated cells. (B) *Escherichia coli* NEB5α, BL21 and MG1655 cells were transformed with pTJV1Sc-rpoB and the RpoB_P564L_ frequencies were determined by plating on LB + rifampicin. (C) *Escherichia coli* MG1655::tetAR was transformed with pTJV1Sc-rpoB, pTJV1Sc-tetA and pTJV1Sc-ackA. Frequencies of the RpoB_P564L_, TetA_*70Y_ and AckA_E54*_ mutations were determined by plating cells on LB + rifampicin, tetracycline and chloroacetate, respectively. (D) Sequential mutation of distinct loci was performed by transforming *E. coli* MG1655 with pTJV1Sc-rpoB, determining recombination efficiency by plating on LB + rifampicin, curing pTJV1Sc-rpoB by growing cells at 42°C, then transforming once more with pTJV1Sc-ackA. Double mutant frequency was assessed by plating on LB + rifampicin + sodium chloroacetate. Averages are based on three independent replicates. Error bars represent SEM. Statistical significance is denoted by asterisks (** = *P*-value < 0.01; *** = *P*-value <0.001; two-tailed Student’s *t*-test of log-transformed values).

We next investigated whether our improved *in vivo* recombineering system could be combined with CRISPR/Cas9 counterselection against wild-type cells for a highly efficient genome editing system. The pCas9CR4 plasmid has *cas9* under the control of an inducible P*_tet_* promoter that was engineered to enable co-maintenance with a genome targeting sgRNA until induction with anhydrotetracycline (aTc) (27). We first transformed pCas9CR4 into the *E. coli* strains NEB5α, BL21-DE3 and MG1655, and subsequently transformed the pTJV1Sc-rpoB editing vector, which possessed an sgRNA targeted to wild-type *rpoB*. After recovery, the cells were transferred to LB broth with spectinomycin and chloramphenicol to select for both plasmids and grown overnight. The following morning, the cultures were then passed into media with or without aTc and grown an additional overnight for induction of Cas9 expression and killing of unedited cells. Cells were then spotted onto plates with and without rifampicin to directly select for the *rpoB* mutation and assess total viable cells, respectively (Figure 2A). The recombination efficiency was between 1 × 10^–5^ and 3 × 10^–3^ without induction of Cas9 expression for all three strains. However, when *cas9* was induced, the average efficiency was ∼5% for BL21 and 60–80% for NEB5α and MG1655, indicating a substantial reduction in wild-type cells (Figure 2B).

To demonstrate that our system could mutagenize different targets, we examined the recombination efficiency of *tetA* and *ackA*. We inserted the *tetA* encoded tetracycline efflux pump that possessed a premature stop codon of about 200 bp from the start codon into MG1655 using traditional λ-Red recombineering (21). Our experiments then mutated the stop codon back to a sense codon, creating the TetA_*70Y_ mutation to enable the full-length translation of *tetA* and confer tetracycline resistance to the cells. We obtained tetracycline-resistant colonies with efficiencies of 5.4 × 10^–4^ and 1.8 × 10^–5^ with and without Cas9 counterselection, respectively, much lower than those observed for *rpoB* (Figure 2C). Next, the acetate kinase gene, *ackA*, was mutated with three nucleotide point mutations to create a premature stop codon (AckA_E54*_) so that the cells are unable to metabolize acetate and are thus resistant to the toxic acetate analog chloroacetate. A recombination efficiency of 5.6% without Cas9 counterselection and over 100% with induction of Cas9 was obtained, as slightly more colonies were observed on the selection plate than on the nonselective plate on average (Figure 2C). These experiments show that targeted counterselection of unedited cells with Cas9 can successfully enrich for recombinants across distinct loci.

The pCas9CR4 plasmid used in these experiments was designed for tight repression of *cas9* and an ssrA degradation tag decreases Cas9 stability, enabling co-maintenance of both genome targeting sgRNA template and *cas9* on two plasmids. Although induction of *cas9* is required for cell death by double-strand break (DSB), we questioned whether some transient level of Cas9 expression also enriches for recombinants, thus we performed experiments to compare the number of mutants obtained with the SCRIBE system in the presence and absence of pCas9CR4. More recombinant cells were produced for all three targets with cells harboring pCas9CR4 than cells without, even without induction of *cas9* expression (Figure 2C). In fact, we were unable to obtain tetracycline-resistant cells when using the pTJV1Sc-tetA plasmid alone. These results suggest that even low levels of *cas9* expression in the uninduced state can increase recombination efficiency possibly by slowing the growth of wild-type cells that must repair DSB or because this low-level DSB stimulates recombination.

The proposed mechanism of ssDNA recombineering asserts that allelic replacement occurs at the replication fork, where the supplied ssDNA replaces an Okazaki fragment on the lagging strand. This mechanism results in a lagging strand bias, where recombination efficiencies for oligos targeting the lagging strand are higher than those targeting the leading strand. To assess whether *in vivo* produced ssDNA proceeds through the same mechanism, we targeted the leading strand of *rpoB* for editing (Supplementary Figure S3A). Surprisingly, this resulted in a similar efficiency as targeting the lagging strand (Supplementary Figure S3B). We wondered whether this was a function of the *in vivo* generation of ssDNA or if the same would be true of the *rpoB* target using traditional λ-Red recombineering. We constructed a control plasmid in which the *msr-msd* and RT coding sequences were removed (pTV1β-rpoB), resulting in constitutive expression of Beta only and sgRNA targeting *rpoB*. We then performed a traditional recombineering experiment by transforming exogenous ssDNA into cells with this plasmid and pCas9CR4 and observed that the lagging oligo was much more efficient, consistent with previous observations (7, 9, 29) and the proposed mechanism of ssDNA recombineering (Supplementary Figure S3C). To evaluate whether the same phenomenon is true of other targets using SCRIBE, we targeted the leading strand of *tetA* with the *in vivo* system (Supplementary Figure S3A). In this case, over 1000-fold fewer recombinant cells were obtained than when targeting the lagging strand (Supplementary Figure S3D). While we suspect that recombineering with *in vivo* ssDNA occurs at the lagging strand of the replication fork, our data suggest that the constant availability of ssDNA may be sufficient to overcome the lagging strand bias in some cases or that an alternative mechanism may influence recombination rates at some locations.

### 3.3 Iterative mutagenesis of two targets

Above all, the high efficiency of mutagenesis found at our target sites confirms a robust system for genome editing, precluding the need to screen large numbers of colonies to identify mutants. To demonstrate that our system could iteratively construct genome modifications, cells that were mutated at the *rpoB* locus were grown at 42°C to cure the cells of pTJV1Sc-rpoB. Subsequent transformation with pTJV1Sc-ackA enabled mutation of the *ackA* gene and produced cells resistant to both rifampicin and chloroacetate (Figure 2D). Amplification and Sanger sequencing of the *rpoB* and *ackA* loci showed that both intended mutations were made, confirming that iterative use of the system could edit multiple loci independently (Supplementary Figure S4).

### 3.4 Co-Expression of dominant-negative *mutL*

**Figure 3.**
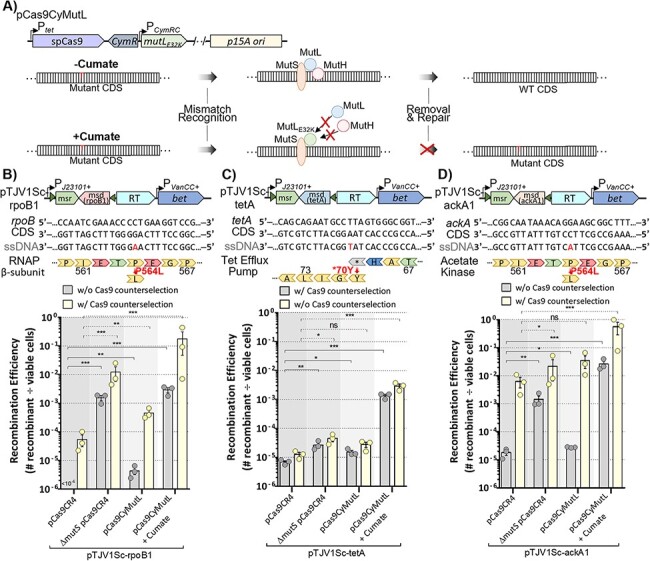
Expression of dominant-negative MutL allele enhances recombination efficiency for single nucleotide point mutations. (A) The MutL_E32K_ gene was cloned into the pCas9CR4 plasmid under the control of the P_CymRC_ promoter for inducible expression. When cumate is added, MutL_E32K_ is expressed and competes with the native MutL for binding to MutS during mismatch repair. The E32K mutation prevents MutH binding, inhibiting removal and repair of the mismatched base. Cells harboring pCas9CyMutL were transformed with (B) pTJV1Sc-rpoB1, (C) pTJV1Sc-tetA or (D) pTJV1Sc-ackA1 encoding single point mutations for generating the RpoB_P564L_, TetA_*70Y_ and AckA_E54*_ mutations, respectively, and grown in liquid culture with or without cumate. The unmodified pCas9CR4 in both wild-type *E. coli* and a *mutS* mutant were used as controls. Recombination efficiency with and without Cas9 counterselection was assessed by plating on LB + rifampicin, LB + tetracycline or M9 + chloroacetate. Data reported are the average of three independent replicates. Error bars represent SEM and statistical significance relative to pCas9CR4 is denoted by asterisks (ns = not significant; * = *P*-value < 0.05; ** = *P*-value < 0.01; *** = *P*-value <0.001; one-way analysis of variance and Tukey’s test of log-transformed values).

Recombineering with ssDNA results in transient production of heteroduplex DNA that the methyl-directed mismatch repair (MMR) system can repair, thus decreasing the observed rate of mutagenesis. Several strategies are known to decrease the rate of MMR and enable more efficient mutagenesis (15, 17, 30–32). For example, single bp mismatches are repaired with different efficiencies, while C:C mismatches evade repair entirely and prevent repair of mismatches within 3 bp (17, 31). The introduction of several adjacent mismatches also prevents repair by MMR (15). However, these strategies present design constraints that cannot always be followed. An alternate strategy is the recombinant expression of negative *mutS* or *mutL* variants that are dominant over wild-type enzymes and prevent efficient repair (32). Accordingly, we cloned *mutL* with the dominant negative E32K mutation into the pCas9CR4 plasmid under the control of a cumate inducible promoter (22) to maintain a two-plasmid system with independent control of *cas9* and *mutL* (Figure 3A). To test this system, we introduced a single bp mismatch of A:C in *rpoB* with and without induction of the negative *mutL*. In cells harboring pCas9CyMutL with *mutL* in the off-state, the average frequency of rifampicin-resistant mutants was 4.9 × 10^–6^ without Cas9-induction and 4.5 × 10^–4^ with Cas9 counterselection (Figure 3B). In contrast, the induction of *mutL* enabled *rpoB* mutations with an average recombination efficiency of 0.2% and 18% without and with Cas9 counterselection (Figure 3B). We also re-examined the *tetA* reversion assays, which introduced a single bp T:T mismatch that is less efficiently repaired than the A:C mismatch used for *rpoB*. Induction of the mutator allele increased the recombination efficiency by about 100-fold both with and without Cas9 counterselection, achieving a maximum efficiency of 0.3% on average (Figure 3C). When generating a single A:G mismatch in the *ackA* gene, *mutL* induction improved recombination by 1000- and 100-fold with and without Cas9 counterselection, respectively, resulting in an average maximum efficiency of 56% (Figure 3D). These results further highlight the versatility of our system in generating precise mutations across a diverse range of loci.

To further illustrate the efficiency enhancement these tools offer over previous methods, we performed the same experiments using the no-SCAR method described previously (12). Single-stranded editing oligos targeting the lagging strand were used to introduce the same single bp mismatches as before in cells harboring pCas9CyMutL and pKDsgRNA, a plasmid encoding the λ-Red proteins Exo, Beta and Gam under the control of the arabinose-inducible P_BAD_ promoter, as well as an sgRNA targeting the wild type gene sequences. When targeting *rpoB*, induction of *mutL* before transformation, during recovery, or both achieved similarly high recombination rates as the retron system, yielding average efficiencies >2% with direct selection on rifampicin and 100% with Cas9 counterselection, a 20-fold increase in recombinants than without *mutL* (Supplementary Figure S5A). Regardless of whether *mutL* was induced, Cas9 counterselection effectively removed unedited cells with 95% of cells plated on aTc being rifampicin-resistant even without *mutL* expression. On the contrary, when targeting ackA, *mutL* induction improved the recombination efficiency less than 10-fold, yielding a maximum efficiency of 4.8 × 10^–4^ when plated on chloroacetate (Supplementary Figure S5B). However, sequencing the *ackA* gene of several putative mutant colonies on the chloroacetate selection media revealed none had acquired the intended mutation. Furthermore, Cas9 counterselection did not substantially enrich for mutants, with <5% of the colonies on the aTc-induced plate able to grow with chloroacetate when patched (Supplementary Figure S5B), indicating most of the colonies on the counterselection plate escaped Cas9 killing. These results are consistent with previous studies examining the rate of Cas9 escape in bacteria, which report frequencies ranging from ∼10^−3^ to 10^−4^ in multiple species (11, 27, 33, 34). Overall, the combined *mutL* and retron-based systems result in consistently high mutagenesis rates for single bp edits and can outperform traditional oligo recombineering methods for certain target loci.

Hindrance of MMR increases the frequency of background genome mutations (35, 36); however, the inducibility of our system minimizes the amount of time in which MMR is inhibited. Similarly, uncontrolled expression of the λ-Red genes has also been shown to increase the rate of spontaneous mutations (37). As such, constitutive expression of *bet* in our system could conceivably produce unintended recombination events in the cell. To assess the background rate of mutagenesis in cells with our SCRIBE system, we measured spontaneous rifampicin resistance (38, 39). Cells were grown overnight, diluted 1:10, grown overnight and then spotted on rifampicin to assess the mutation frequency. The wild-type cells spontaneously acquired rifampicin resistance at a frequency of 6 × 10^–7^ on average. Cells constitutively expressing *bet* and cells with the uninduced pCas9CyMutL did not differ significantly from the wild-type (Supplementary Figure S6A). However, induction of the mutator *mutL* resulted in an average mutation frequency of about 1.3 × 10^–5^, an ∼20-fold increase over the wild-type (Supplementary Figure S6A).

While these results indicate that the unexpressed *mutL* and constitutive *bet* did not increase the rate of point mutations, we wanted to assess further whether other genomic mutations and rearrangements that would not manifest as rifampicin resistance were present. Accordingly, we performed whole-genome sequencing on the strains grown for two overnights to identify polymorphisms within the entire population. A complete list of the mutations identified is in Supplemental File 2. Our analysis revealed 41 total mutations in the population constitutively expressing *bet,* constituting a mutation rate of 5.9 × 10^–11^ mutations per cell per generation (Supplementary Figure S6B), with one new IS5 mobile element insertion into the *rclA* gene. The uninduced pCas9CyMutL cells displayed a slightly higher mutation rate of 2.4 × 10^–10^; however, both rates were similar to the wild-type control, which exhibited a mutation rate of 1.0 × 10^–10^. In contrast, 478 mutations were found in the population with *mutL* induced for a mutation rate of 1.59 × 10^–9^ mutations per cell per generation (Supplementary Figure S6B), as well as a deletion of the ∼1200 bp *insH21* mobile element. Even still, this low frequency of background mutations indicates selecting a mutant with unintended mutations would be a rare occurrence. Overall, these results demonstrate that maintenance of plasmids with the *mutL* mutator allele or constitutive expression of *bet* do not increase the frequency of background mutations to a level that would cause concern from a genetic engineering standpoint.

### 3.5 Alternative recombinases enhance efficiency for some targets

**Figure 4.**
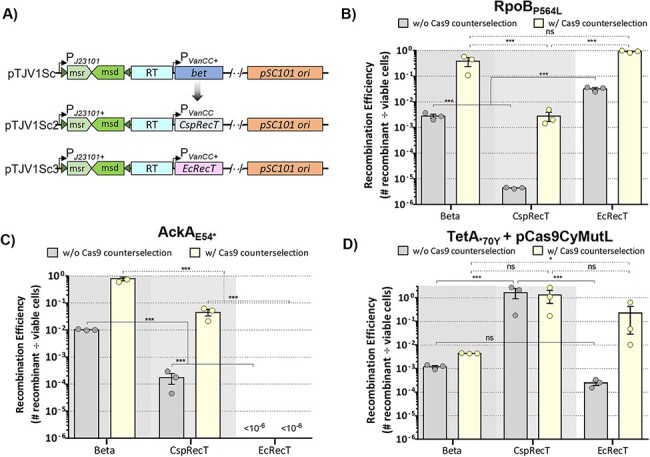
Alternative recombinases can enhance efficiency for some targets. (A) The *bet* CDS on pTJV1Sc was replaced with genes encoding the CspRecT and EcRecT recombinases. Recombination efficiency for each of the recombinase variants with and without induction of *cas9* expression was assessed for (B) the RpoB_P564L_ mutation by plating cells on LB + rifampicin, (C) the AckA_E54*_ mutation by plating cells on LB + chloroacetate and (D) the TetA_*70Y_ mutation by plating cells on LB + tetracycline. The pCas9CyMutL plasmid was used for the *tetA* experiments. Data reported are the average of three independent replicates. Error bars represent SEM and statistical significance is denoted by asterisks (ns = not significant; *** = *P*-value <0.001; one-way analysis of variance and Tukey’s test of log-transformed values).

Recently, Wannier *et al.* identified *recT* from a *Collinsella stercoris* phage (CspRecT) with improved recombination efficiency compared to Beta for oligo mediated recombineering in *E. coli* (40). To examine the effect of alternative recombinases in our system, we replaced *bet* with CspRecT, as well as EcRecT from the *E. coli* BL21-DE3 chromosome (Figure 4A). We then compared the efficiency of these plasmids across three different targets. Surprisingly, our results showed inconsistency for which DNA-binding protein promoted the highest number of recombinants between the different targets. For the *rpoB* mutation, EcRecT was more efficient than both Beta and CspRecT, with 92% efficiency when using Cas9 counterselection (Figure 4B). On the contrary, the *ackA* mutation was more efficient with Beta, where 80% of cells were mutants compared to 4.5% for CspRecT and no *ackA* mutants with EcRecT (Figure 4C). Given the improvement in efficiency seen for *tetA* when expressing MutL_E32K_, we used pCas9CyMutL in combination with the alternative recombinases. We obtained over 100% efficiency for the *tetA* reversion with CspRecT, both with and without Cas9 counterselection. Although slightly more colonies were observed on the tetracycline plate than on the nonselective plate for two of the replicates (Figure 4D), the CspRecT clearly outperformed Beta and EcRecT where relatively low levels of mutants were found. Thus, in circumstances where a mutation is difficult to obtain, it is advisable to try these alternative recombinases, while the mechanism causing these discrepancies warrants further investigation.

## 4. Discussion

In moving the SCRIBE machinery onto the temperature-sensitive pSC101 origin of replication and combining it with Cas9 counterselection, we successfully constructed an *in vivo* system for ssDNA recombineering that is highly efficient and iterative. Targeted counterselection using CRISPR/Cas9 enabled efficiencies near 100% after a single night of outgrowth post-transformation, reducing the number of colonies needing to be screened for mutant identification. Moreover, both the ssDNA template and sgRNA sequence can be retargeted for modification of any genomic locus with an appropriate PAM in a single cloning step using sequence overlap cloning methods. Numerous loci can be mutated without the need for orthogonal selection markers or extensive screening procedures, increasing the speed and ease of generating desirable mutants. In addition, the pCas9CR4 and pCas9CyMutL plasmids can be cured using pKDsgRNA-p15 (Addgene #62656) as previously described (12), yielding plasmid-free mutants for downstream applications.

Recombineering is an important tool in the biological sciences, and continued enhancement of recombination efficiency across diverse target loci will decrease strain construction times. Recently, Lopez *et al.* showed that increasing the abundance of ssDNA produced by retrons significantly improves recombination efficiencies (41), consistent with the enhanced efficiencies we observed when increasing the msr–msd/RT expression and *bet* through promoter modifications. A complementary strategy is to reduce the rate of ssDNA degradation by host nucleases, as the High-efficiency SCRIBE (HiSCRIBE) system by Farzadfard *et al.* (20) describes. This system improves upon their original SCRIBE design by coupling a strong ribosome-binding site for *bet* on the SCRIBE plasmid with CRISPRi-enabled transcriptional interference of the endogenous exonucleases. Recombination efficiencies of nearly 100% for *galK* and ∼25% for a kanamycin resistance gene target were obtained with these improvements. However, employing the nuclease-deficient dCas9 for CRISPRi prevents the use of the nuclease-active Cas9 for counterselection. Although the authors show that CRISPR/Cas9 counterselection can select for recombinant cells at high efficiencies in an exonuclease knockout strain, this strategy prevents easy system portability since a mutant strain must be used.

Lim *et al.* (42) also describe a CRISPR/retron-based editing system enabling trackable editing and functional screening across multiple loci in a population. However, this system requires the sequential transformation and maintenance of three plasmids. Moreover, the construction of their retron/sgRNA plasmid requires multiple enzymatic digestions for cloning, contrary to our two-plasmid system, which simultaneously retargets the retron and sgRNA in a single two-fragment cloning step. Most importantly, both the HiSCRIBE and the CRISPR/retron system of Lim *et al.* (20, 42) would require multiple orthogonal selection markers to mutate multiple loci within a single cell, as no easy method of plasmid curing is included in either design, preventing iterative mutagenesis. Although the recombination efficiencies achieved with our system in the absence of counterselection are less than those reported for HiSCRIBE, Cas9-induced cell death of unedited cells in the population enabled apparent efficiencies on par with HiSCRIBE. The ease with which our system can be retargeted and used iteratively to incorporate multiple edits combined with its high efficiency in mutant selection provides a quick and straightforward method for constructing mutants relevant to both research and biotechnological applications. Methods enabling multiplexed editing of numerous genomic loci within populations and continuous evolution of targeted loci, using retron-mediated recombineering, have been recently described (20, 42–44) and rely on efficient editing for generating a library of variants. Our system would be well suited for constructing such libraries, with the added advantage of being curable, thus enabling editing of distant loci within the same cell through repeated rounds of library recombineering.

## Supplementary Material

ysac007_Supp

## Data Availability

Raw sequence reads for the off-target mutation frequency experiments are available under the BioProject ID PRJNA813865 in the NCBI databases.
