# Supplementary material for: Efficient and iterative retron-mediated in vivo recombineering in Escherichia coli
Source: Synth Biol (Oxf). 2022 May 3;7(1):ysac007. doi: 10.1093/synbio/ysac007 (PMC9165427; doi:10.1093/synbio/ysac007)
Supplement: ysac007_Supp [file ysac007_supp.zip › Supplemental File 1.pdf]

Supplemental Information for

Efficient and iterative retron-mediated in vivo recombineering in E. coli

Adam J. Ellington & Christopher R. Reisch*

*Corresponding Author
Email: creisch@ufl.edu

This section includes:

Tables S1-S2
Figures S1-S6

Other supplemental materials for this manuscript include the following:

Supplemental File 2
PlasmidSeqs.zip
Primers_GeneFrags.fa

Table S1: List of strains used in this work

STRAIN	GENOTYPE	USED IN
Escherichia coli NEB5α	*fhuA2 Δ(argF-lacZ)U169 phoA glnV44 Φ80 Δ(lacZ)M15 gyrA96 recA1 relA1 endA1 thi-1 hsdR17*	Figure 1, 2
Escherichia coli K-12 MG1655	*F– λ– ilvG– rfb-50 rph-1*	Figure 2, 3, 4, S3, S5, S6
Escherichia coli BL21(DE3)	*F– ompT gal dcm lon hsdSB(rB–mB–) λ(DE3 [lacI lacUV5-T7p07 ind1 sam7 nin5]) [malB+]K-12(λS)*	Figure 2

Table S2: List of plasmids used in this work

NAME	DESCRIPTION	CONSTRUCTION METHOD	REFERENCE
pFF745	Original SCRIBE plasmid	-	(Farzadfard & Lu, 2014)
pTLlSc	pKDsgRNA vector with functional components of SCRIBE and sgRNA targeting *E. coli rpoB.*	The Ec86 retron cassette/beta recombinase CDS and pKDsgRNA vector were PCR amplified with primers 2215/2216 and primers 25/1530, respectively, followed by assembly via Gibson assembly	This work
pTLlSc-rpoB	pTLlSc re-targeted for *rpoB* to create the CCT→TTG triple point mutation and generate $\mathrm{RpoB}_{\mathrm{P564L}}$.	Primers 2107 and 2108 were used to amplify pTLlSc and re-target the msd for the *E. coli rpoB* gene, followed by assembly via Gibson assembly	This work; Figure 1
pTJSc-rpoB	pTLlSc-rpoB with P_{J23101} replacing P_{lacO} for strong, constitutive expression of Ec86 retron cassette.	Primers 2234/2235 and 1535/449 were used to amplify BBa_J23101 (iGEM Registry of Standard Biological Parts) and pTLlSc-rpoB, respectively, followed by Gibson assembly	This work; Figure 1
pTLlVSc-rpoB	pTLlSc-rpoB with P_{VanCC} added upstream of *bet* for strong constitutive expression.	Primers 2239/2240 and 2238/643 were used to amplify the P_{VanCC} promoter and pTLlSc-rpoB, respectively, followed by Gibson assembly	This work; Figure 1
pTJVSc-rpoB	pTLlSc-rpoB with P_{J23101} replacing P_{lacO} for constitutive expression of Ec86 retron cassette and P_{VanCC} added upstream of *bet* for strong constitutive expression.	Primers 2239/2240 and 2238/643 were used to amplify the P_{VanCC} promoter and pTJSc-rpoB, respectively, followed by Gibson assembly	This work; Figure 1, S1
pTJV1Sc-rpoB (Addgene #166978; NCBI Acc. OK377063)	pTJVSc-rpoB with optimized P_{J23101} and P_{VanCC} promoter sequences for improved recombineering efficiency	Primers 2326/2358 and 2359/2325 were used to amplify pTJVSc-rpoB and introduce variation in the -10 and -35 elements of the P_{J23101} and P_{VanCC} promoters, followed by Gibson assembly	This work; Figure 1, 2, 4, S1, S3
pTJV1Sc-tetA	Targets chromosomally integrated *tetA* gene for reversion of premature stop codon via single point mutation, TAA→TAT, restoring tetracycline resistance	Primers 2564/2605 and 2565/2606 were used to amplify pTJV1Sc-rpoB and re-target the msd and sgRNA for *tetA*, followed by Gibson assembly	This work; Figure 2, 3, 4, S3

NAME	DESCRIPTION	CONSTRUCTION METHOD	REFERENCE
pTJV1Sc-ackA	Optimized SCRIBE construct targeting the *ackA* gene for incorporating the CAGG→GACT triple point mutation to generate a premature stop codon.	Primers 2452/93 and 2453/92 were used to amplify pTJV1Sc-rpoB and re-target the msd and sgRNA for *ackA*, followed by Gibson assembly	This work; Figure 2, 4
pTJV1Sc-rpoB1	Optimized SCRIBE construct targeting *rpoB* to create C→T single point mutation and generate $RpoB_{P564L}$.	Primers 2634 & 2635 were used to amplify pTJV1Sc-rpoB to modify the msd encoding the *rpoB* CCT→TTG triple point mutation to a CCT→CTT single point mutation, followed by Gibson assembly	This work; Figure 3
pTJV1Sc-ackA1	Optimized SCRIBE construct targeting *ackA* to create G→T single point mutation and generate $AckA_{E54*}$.	Primers 2935 & 2936 were used to amplify pTJV1Sc-ackA followed by Gibson assembly	This work; Figure 3
pTJV1Sc2-rpoB	pTJV1Sc-rpoB with *bet* replaced by the CspRecT recombinase	Primers 2673/2674 were used to amplify pTJV1Sc-rpoB followed by Gibson assembly with the CspRecT gBlock (Integrated DNA Technologies)	This work; Figure 4
pTJV1Sc2-tetA (Addgene #166982; NCBI Acc. OK423785)	pTJV1Sc-tetA with *bet* replaced by the CspRecT recombinase	Primers 2605/2673 and 2606/2674 were used to amplify pTJV1Sc-tetA followed by Gibson assembly with the CspRecT gBlock (Integrated DNA Technologies)	This work; Figure 4
pTJV1Sc2-ackA	pTJV1Sc-ackA with *bet* replaced by the CspRecT recombinase	Primers 2453/92 and 2452/93 were used to amplify pTJV1Sc2-rpoB to re-target the msd and sgRNA for ackA followed by Gibson assembly	This work; Figure 4
pTJV1Sc3-rpoB (Addgene #166983; NCBI Acc. OK483345)	pTJV1Sc-rpoB with *bet* replaced by the EcRecT recombinase	Primers 2732/2733 were used to amplify EcRecT from the *E. coli* BL21 chromosome. and pTJV1Sc-rpoB, respectively, followed by Gibson assembly	This work; Figure 4
pTJV1Sc3-tetA	pTJV1Sc-tetA with *bet* replaced by the EcRecT recombinase	Primers 2564/2605 and 2565/2606 were used to amplify pTJV1Sc3-rpoB, followed by Gibson assembly	This work; Figure 4
pTJV1Sc3-ackA	pTJV1Sc-ackA with *bet* replaced by the EcRecT recombinase	Primers 2452/93 and 2453/92 were used to amplify pTJV1Sc3-rpoB, followed by Gibson assembly	This work; Figure 4

NAME	DESCRIPTION	CONSTRUCTION METHOD	REFERENCE
pTJV1Sc-rpoB_ld	pTJV1Sc targeting the leading strand of *rpoB*	Primers 2105/2106 were used to amplify pTJV1Sc-rpoB, followed by Gibson assembly	This work; Figure S3
pTJV1Sc-tetA_ld	pTJV1Sc targeting the leading strand of *tetA*	Primers 2564/2605 and 2565/2606 were used to amplify pTJV1Sc-rpoB, followed by Gibson assembly	This work; Figure S3
pCas9CR4	Encodes spCas9 under control of the ATC-inducible P_{Tet} promoter for tight control of expression	-	(Reisch & Prather, 2015 & 2017); Figure 2, 3, 4, S3, S5
pCas9CyMutL (Addgene #166981; NCBI Acc. OK483344)	Cumate-inducible expression of *mutL* with negative E32K mutation	Primers 2698/2699 were used to amplify pCas9CR4. Primers 2154/2155 were used to amplify *cymR*, P_{CymRC}, RiboJ, and *mutLE32K* from pBCyMutL2. Assembly by Gibson assembly.	This work; Figure 3, 4, S5, S6
pKD46	Arabinose inducible expression of λ *exo, bet, and gam* with temperature sensitive origin of replication, pSC101-ts	-	(Datsenko & Wanner, 2000)
pTV1β-rpoB	pTJV1Sc-rpoB with the msr-msd and RT sequences deleted, so only *bet* is expressed for traditional ssDNA recombineering	Primers 2239/156 were used to amplify pTJV1Sc-rpoB followed by blunt ligation	This work; Figure S3
pKDsgRNA	Arabinose inducible expression of λ *exo, bet, and gam* with temperature sensitive origin of replication, pSC101-ts, and constitutively expressed sgRNA	-	(Reisch & Prather, 2015 & 2017)
pKDsgRNA-rpoB	pKDsgRNA with sgRNA targeting the *E. coli rpoB* gene	Primers 468/43 were used to amplify the sgRNA from pTJV1Sc-rpoB. Primers 759/66 and 503/44 were used to amplify pKDsgRNA followed by Gibson assembly of all three fragments	This work; Figure S5
pKDsgRNA-ackA	pKDsgRNA with sgRNA targeting the *E. coli ackA* gene	Primers 92/738 were used to amplify pKDsgRNA followed by blunt ligation	This work; Figure S5

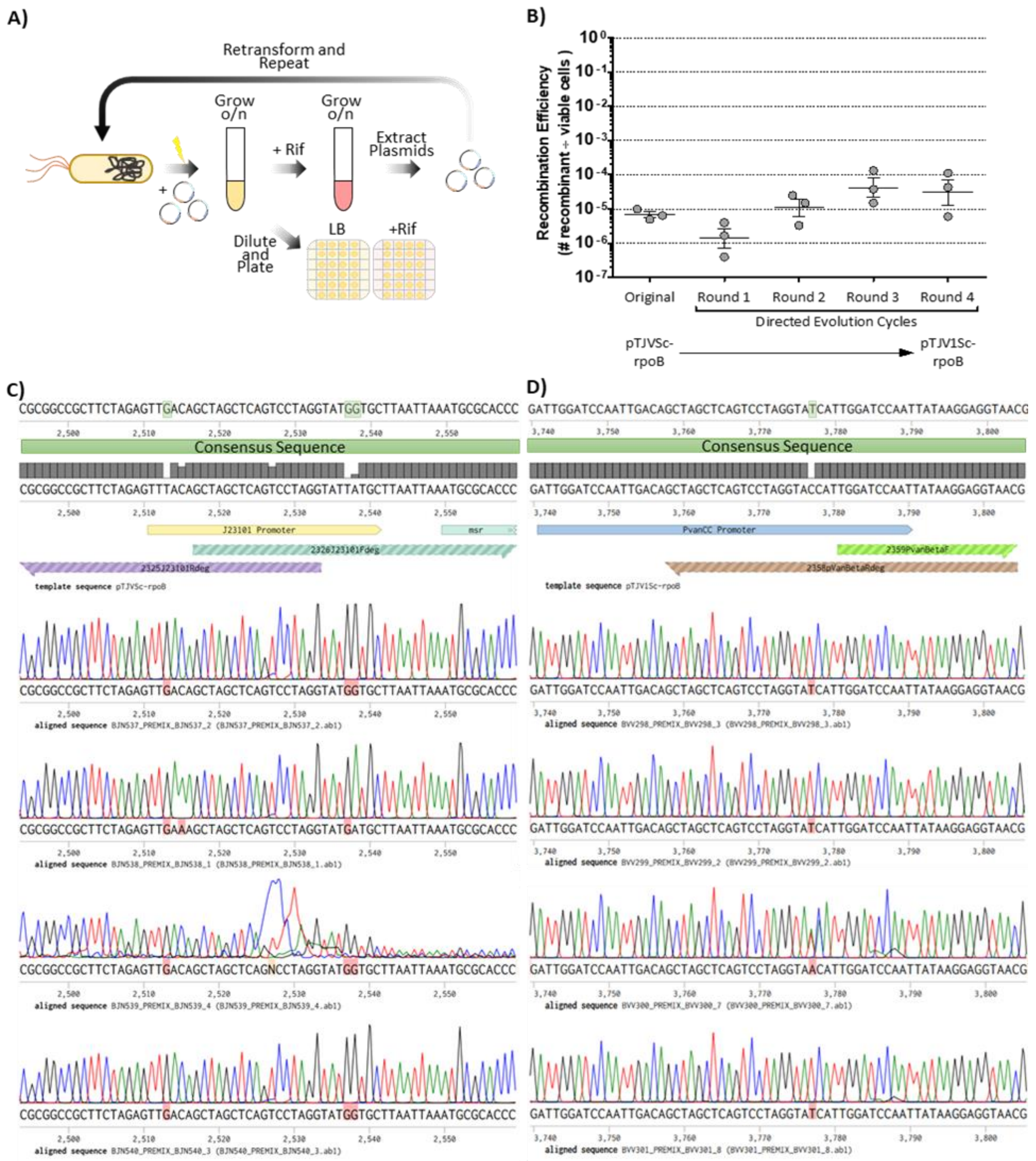

Fig. S1. PCR amplification of pTJVSc-rpoB using degenerate primers produces promoter sequence variants that enhance recombination frequency. (A) Schematic illustrating the directed evolution process for promoter optimization. Cells were transformed with the pTJVSc-rpoB pool of promoter variants. Rifampicin was added after overnight growth to select for recombinant cells. Plasmids were then extracted from the pool and retransformed into fresh cells. This process was repeated four times with samples taken during each round to assess the recombination frequency of the plasmid pool. **(B)** Recombination efficiency of the pTJVSc-rpoB pool after each round of selection (Error bars represent SEM for three independent samples). Alignment of **(C)** P_{J23101} promoter sequences and **(D)** P_{VanCC} promoter sequences from four randomly selected colonies after the fourth round of selection.

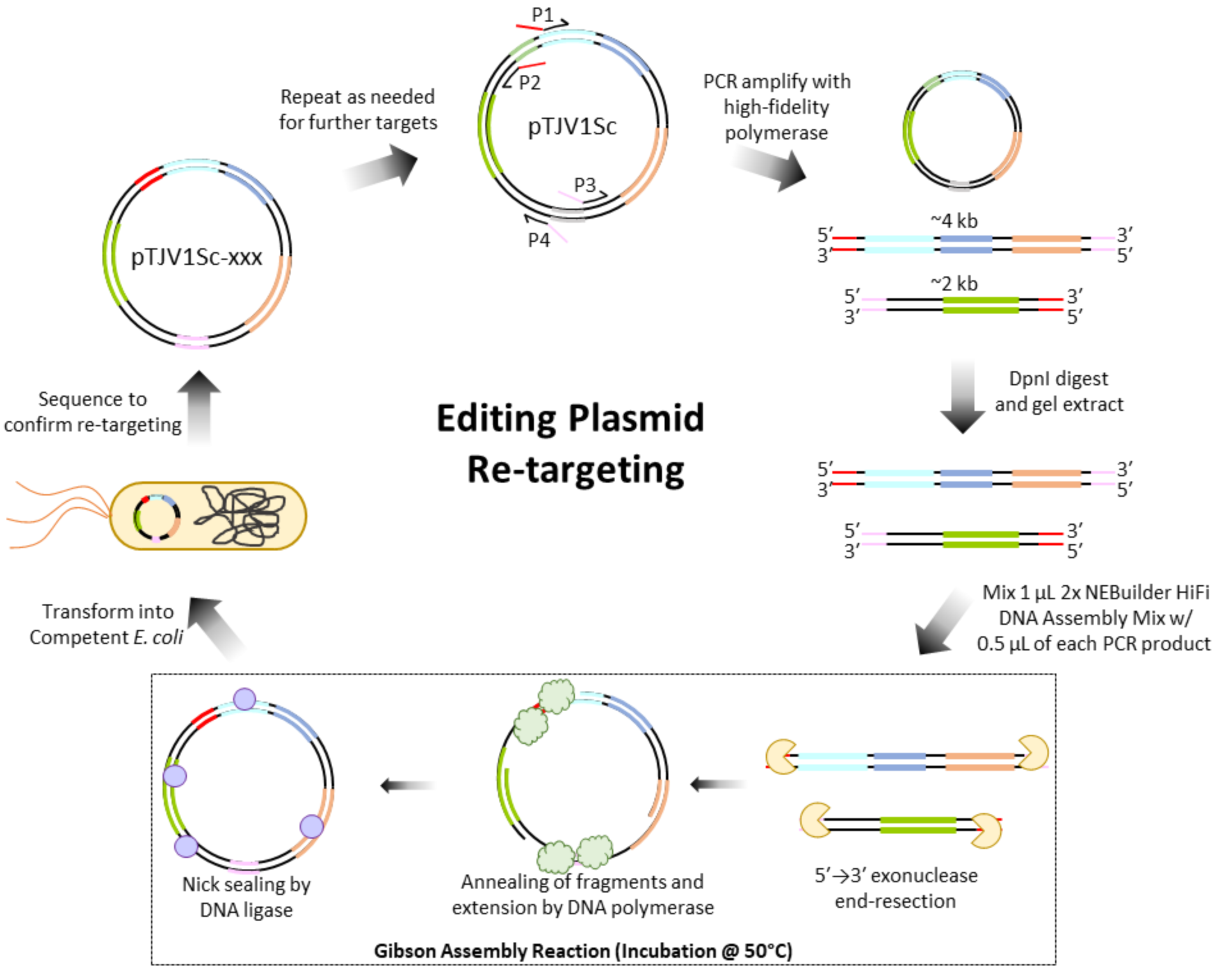

Figure S2: Simultaneous retargeting of *msd* and sgRNA on pTJV1Sc by Gibson assembly cloning. The pTJV1Sc plasmid is PCR amplified using primers P1 and P3 in one reaction and P2 and P4 in a separate reaction. P1 and P2 contain 5′ overhangs encoding the new *msd* sequence and the P3 and P4 overhangs encode the new sgRNA sequence. After amplification, samples are DpnI-treated to remove the plasmid template and the ~4 kb and ~2 kb fragments are gel extracted from a 0.8% agarose gel. PCR products are mixed with the Gibson reagents and incubated at 50°C. The exonuclease (yellow) degrades the ends of the DNA fragments in the 5′→3′ direction generated single-stranded overhangs. The complementary overhangs from each fragment are annealed together and a DNA polymerase (green) extends the annealed fragments to fill in the gaps. Finally, DNA ligase (purple) seals the remaining nick, resulting in a completed plasmid. The entire Gibson reaction is transformed into competent *E. coli* cells and clones are screened by sequencing to confirm successful retargeting of the *msd* and sgRNA sequences.

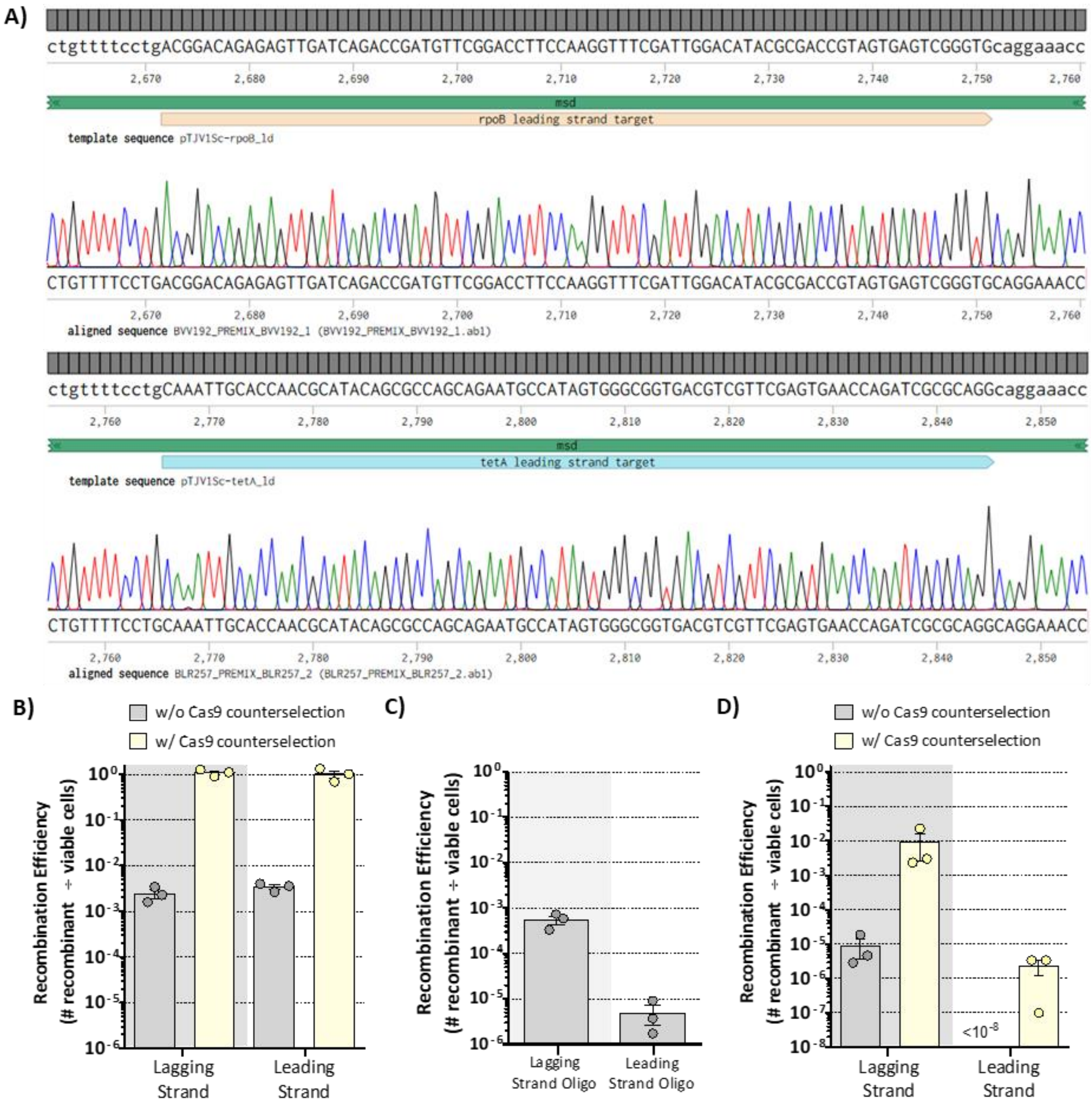

Fig. S3. Effect of targeting the leading vs. lagging strand with *in vivo* recombineering. (A) pTJV1Sc-rpoB and pTJV1Sc-tetA were modified to target the leading strand of their respective targets, creating pTJV1Sc-rpoB_ld and pTJV1Sc-tetA_ld. Successful retargeting was verified by Sanger sequencing. **(B)** Cells were transformed with pTJV1Sc-rpoB and pTJV1Sc-rpoB_ld. Recombination efficiencies both with and without induction of *cas9* were determined by plating on rifampicin. **(C)** Cells harboring pTV1β were transformed with oligos targeting the lagging and leading strand of *rpoB*. The $RpoB_{P564L}$ frequencies were determined as described in [A]. **(D)** Cells were transformed with pTJV1Sc-tetA and pTJV1Sc-tetA_ld. $TetA_{*70Y}$ frequencies both with and without induction of *cas9* were determined by plating on tetracycline. Averages based on three independent replicates. Error bars represent standard error.

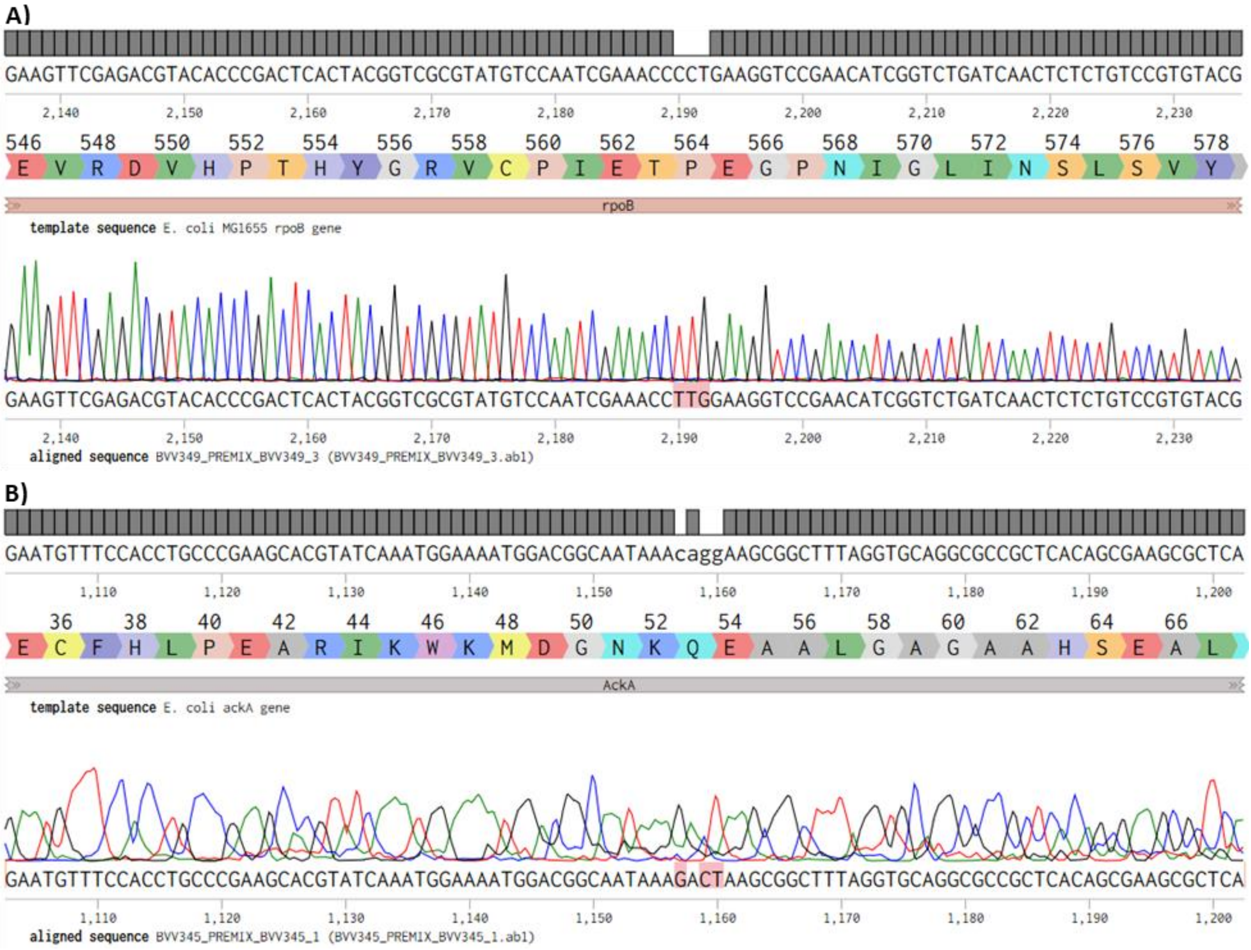

Fig. S4. Iterative editing with pTJV1Sc allows mutation of multiple distinct loci. Sequence alignments for the **(A)** *rpoB* and **(B)** *ackA* genes from a randomly selected recombinant colony.

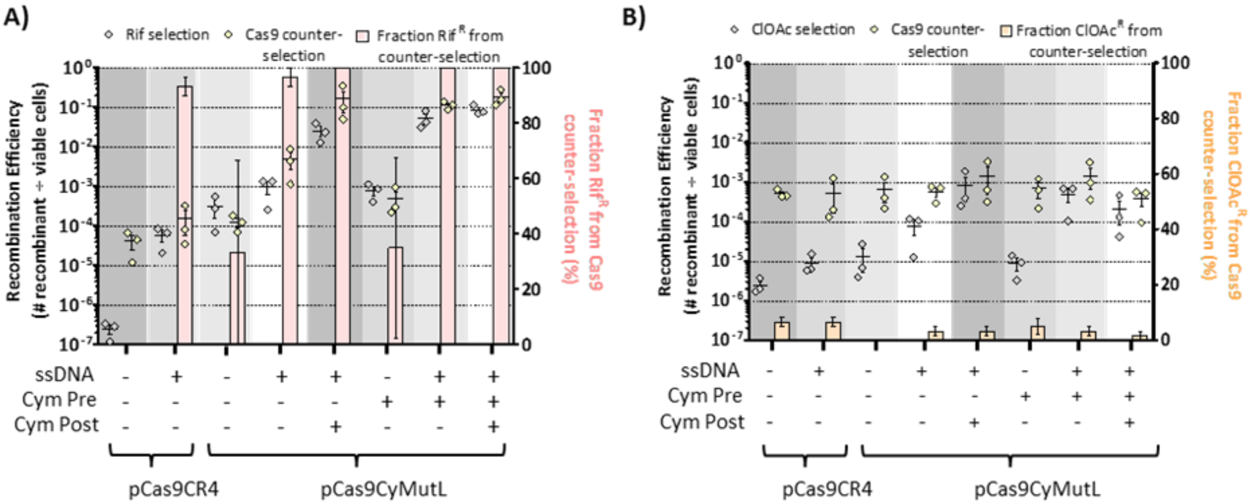

Fig. S5. Oligo-mediated recombineering using pCas9CyMutL and pKDsgRNA can enable single bp mutations for some targets. *E. coli* MG1655 cells harboring pCas9CR4 or pCas9CyMutL and **A)** pKDsgRNA-rpoB or **B)** pKDsgRNA-ackA were transformed with oligos containing single bp mismatches for generating the **A)** $RpoB_{P564L}$ or **B)** $AckA_{E54*}$ mutations. Recombination efficiencies were determined both by plating cells on media containing rifampicin or chloroacetate, respectively, as well as media containing aTc for the induction of cas9 expression. Colonies surviving on the aTc induced plate were then patched onto rifampicin or chloroacetate plates to confirm the intended resistance phenotypes.

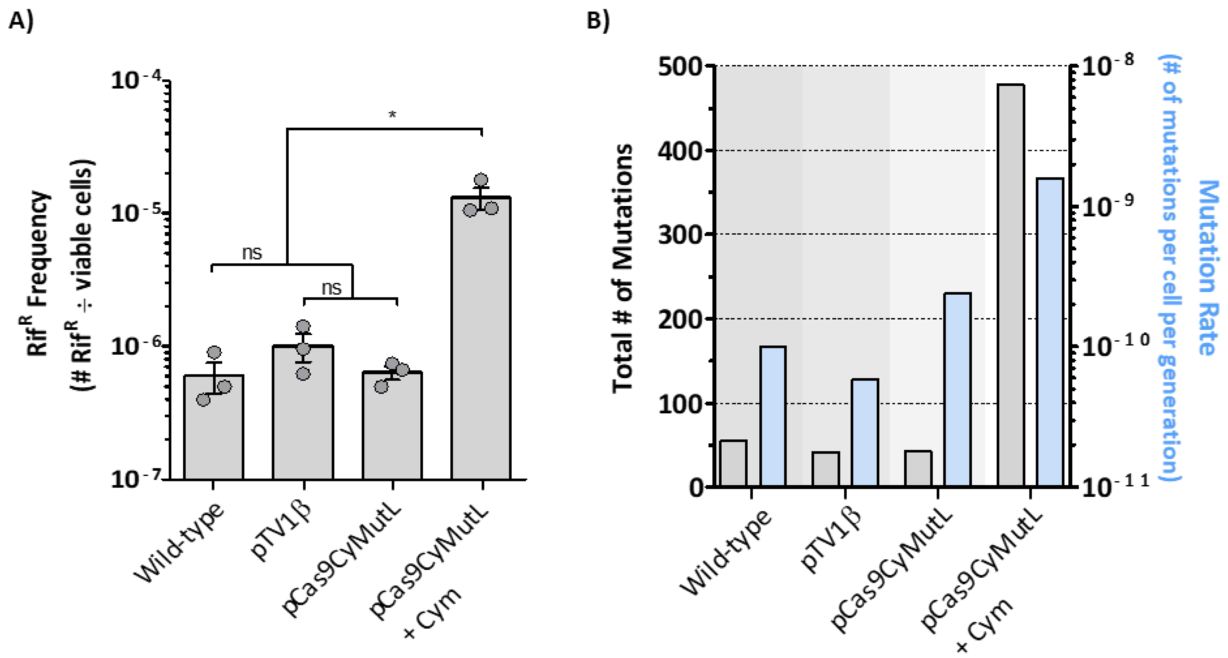

Fig. S6. Analysis of background mutation frequencies in Beta- and MutL$_{E32K}$-expressing cells. (A) Mutation frequencies were assessed for wild-type MG1655 and cells harboring either the Beta-expressing pTJV1β or pCas9CyMutL plasmids by plating on rifampicin after 48 hours of growth (see methods for details). The Rif^R frequency is calculated as the number of mutants divided by the total number of viable cells. Averages are based on three independent replicates. Error bars represent standard error. Statistical significance is denoted by asterisks (ns = not significant; * = P-value < 0.05; two-tailed Welch's T-test). **(B)** Genome-wide mutational events were assessed using whole-genome sequencing to identify polymorphisms within each population. The total number of mutations (gray bars) are displayed on the left axis and mutation rate (blue bars) is shown on the right axis.
