# Supplementary material for: Efficient and iterative retron-mediated in vivo recombineering in Escherichia coli
Source: Synth Biol (Oxf). 2022 May 3;7(1):ysac007. doi: 10.1093/synbio/ysac007 (PMC9165427; doi:10.1093/synbio/ysac007)
Supplement: ysac007_Supp [file ysac007_supp.zip › PlasmidSeqs.zip!/PlasmidSeqs/pCas9CyMutL.gb]

LOCUS       OK483344                9659 bp    DNA     circular SYN 15-APR-2022
DEFINITION  Cloning vector pCas9CyMutL plasmid pCas9CyMutL, complete sequence.
ACCESSION   OK483344
VERSION     OK483344
KEYWORDS    .
SOURCE      Cloning vector pCas9CyMutL
  ORGANISM  Cloning vector pCas9CyMutL
            other sequences; artificial sequences; vectors.
REFERENCE   1  (bases 1 to 9659)
  AUTHORS   Ellington,A.J. and Reisch,C.R.
  TITLE     Direct Submission
  JOURNAL   Submitted (11-OCT-2021) Microbiology and Cell Science, University
            of Florida, 1355 Museum Dr, Gainesville, FL 32611, United States
COMMENT     ##Assembly-Data-START##
            Assembly Method       :: Addgene proprietary assembly program v.
                                     03-2021
            Assembly Name         :: pCas9CyMutL
            Coverage              :: 680x
            Sequencing Technology :: Illumina
            ##Assembly-Data-END##
FEATURES             Location/Qualifiers
     source          1..9659
                     /organism="Cloning vector pCas9CyMutL"
                     /mol_type="other DNA"
                     /db_xref="taxon:2935457"
                     /plasmid="pCas9CyMutL"
                     /note="Addgene:166981"
     gene            join(9270..9659,1..222)
                     /gene="cymR"
     CDS             join(9270..9659,1..222)
                     /gene="cymR"
                     /codon_start=1
                     /transl_table=11
                     /product="HTH-type transcriptional regulator CymR"
                     /protein_id="UOX75814"
                     /translation="MSPKRRTQAERAMETQGKLIAAALGVLREKGYAGFRIADVPGAA
                     GVSRGAQSHHFPTKLELLLATFEWLYEQITERSRARLAKLKPEDDVIQQMLDDAAEFF
                     LDDDFSIGLDLIVAADRDPALREGIQRTVERNRFVVEDMWLGVLVSRGLSRDDAEDIL
                     WLIFNSVRGLVVRSLWQKDKERFERVRNSTLEIARERYAKFKR"
     regulatory      281..308
                     /regulatory_class="terminator"
                     /note="phage T7 early transcription terminator; T7Te
                     terminator"
     regulatory      324..395
                     /regulatory_class="terminator"
                     /note="transcription terminator T1 from the E. coli rrnB
                     gene; rrnB T1 terminator"
     CDS             complement(419..>454)
                     /note="wild-type LAA variant; ssrA tag; (McGinness et al.,
                     2006)"
                     /codon_start=1
                     /transl_table=11
                     /product="C-terminal peptide that mediates degradation in
                     bacteria through the ClpXP and ClpAP proteases"
                     /protein_id="UOX75809"
                     /translation="AANDENYALAA"
     CDS             complement(<455..4558)
                     /note="generates RNA-guided double strand breaks in DNA;
                     Cas9; from the Streptococcus pyogenes Type II CRISPR/Cas
                     system"
                     /codon_start=1
                     /transl_table=11
                     /product="Cas9 (Csn1) endonuclease"
                     /protein_id="UOX75810"
                     /translation="MDKKYSIGLDIGTNSVGWAVITDEYKVPSKKFKVLGNTDRHSIK
                     KNLIGALLFDSGETAEATRLKRTARRRYTRRKNRICYLQEIFSNEMAKVDDSFFHRLE
                     ESFLVEEDKKHERHPIFGNIVDEVAYHEKYPTIYHLRKKLVDSTDKADLRLIYLALAH
                     MIKFRGHFLIEGDLNPDNSDVDKLFIQLVQTYNQLFEENPINASGVDAKAILSARLSK
                     SRRLENLIAQLPGEKKNGLFGNLIALSLGLTPNFKSNFDLAEDAKLQLSKDTYDDDLD
                     NLLAQIGDQYADLFLAAKNLSDAILLSDILRVNTEITKAPLSASMIKRYDEHHQDLTL
                     LKALVRQQLPEKYKEIFFDQSKNGYAGYIDGGASQEEFYKFIKPILEKMDGTEELLVK
                     LNREDLLRKQRTFDNGSIPHQIHLGELHAILRRQEDFYPFLKDNREKIEKILTFRIPY
                     YVGPLARGNSRFAWMTRKSEETITPWNFEEVVDKGASAQSFIERMTNFDKNLPNEKVL
                     PKHSLLYEYFTVYNELTKVKYVTEGMRKPAFLSGEQKKAIVDLLFKTNRKVTVKQLKE
                     DYFKKIECFDSVEISGVEDRFNASLGTYHDLLKIIKDKDFLDNEENEDILEDIVLTLT
                     LFEDREMIEERLKTYAHLFDDKVMKQLKRRRYTGWGRLSRKLINGIRDKQSGKTILDF
                     LKSDGFANRNFMQLIHDDSLTFKEDIQKAQVSGQGDSLHEHIANLAGSPAIKKGILQT
                     VKVVDELVKVMGRHKPENIVIEMARENQTTQKGQKNSRERMKRIEEGIKELGSQILKE
                     HPVENTQLQNEKLYLYYLQNGRDMYVDQELDINRLSDYDVDHIVPQSFLKDDSIDNKV
                     LTRSDKNRGKSDNVPSEEVVKKMKNYWRQLLNAKLITQRKFDNLTKAERGGLSELDKA
                     GFIKRQLVETRQITKHVAQILDSRMNTKYDENDKLIREVKVITLKSKLVSDFRKDFQF
                     YKVREINNYHHAHDAYLNAVVGTALIKKYPKLESEFVYGDYKVYDVRKMIAKSEQEIG
                     KATAKYFFYSNIMNFFKTEITLANGEIRKRPLIETNGETGEIVWDKGRDFATVRKVLS
                     MPQVNIVKKTEVQTGGFSKESILPKRNSDKLIARKKDWDPKKYGGFDSPTVAYSVLVV
                     AKVEKGKSKKLKSVKELLGITIMERSSFEKNPIDFLEAKGYKEVKKDLIIKLPKYSLF
                     ELENGRKRMLASAGELQKGNELALPSKYVNFLYLASHYEKLKGSPEDNEQKQLFVEQH
                     KHYLDEIIEQISEFSKRVILADANLDKVLSAYNKHRDKPIREQAENIIHLFTLTNLGA
                     PAAFKYFDTTIDRKRYTSTKEVLDATLIHQSITGLYETRIDLSQLGGD"
     regulatory      4594..4649
                     /regulatory_class="promoter"
                     /note="overlapping promoters for bacterial tetR and tetA"
     protein_bind    4595..4613
                     /note="bacterial operator O2 for the tetR and tetA genes;
                     tet operator; tetO"
                     /bound_moiety="tetracycline repressor TetR"
     gene            4700..5323
                     /gene="tetR from transposon Tn10"
     CDS             4700..5323
                     /gene="tetR from transposon Tn10"
                     /note="TetR binds to the tetracycline operator tetO to
                     inhibit transcription. This inhibition can be relieved by
                     adding tetracycline or doxycycline.; TetR"
                     /codon_start=1
                     /transl_table=11
                     /product="tetracycline repressor TetR"
                     /protein_id="UOX75811"
                     /translation="MSRLDKSKVINSALELLNEVGIEGLTTRKLAQKLGVEQPTLYWH
                     VKNKRALLDALAIEMLDRHHTHFCPLEGESWQDFLRNNAKSFRCALLSHRDGAKVHLG
                     TRPTEKQYETLENQLAFLCQQGFSLENALYALSAVGHFTLGCVLEDQEHQVAKEERET
                     PTTDSMPPLLRQAIELFDHQGAEPAFLFGLELIICGLEKQLKCESGS"
     regulatory      5353..5455
                     /regulatory_class="promoter"
                     /note="promoter of the E. coli cat gene encoding
                     chloramphenicol acetyltransferase"
     gene            5456..6115
                     /gene="cat"
     CDS             5456..6115
                     /gene="cat"
                     /note="confers resistance to chloramphenicol; CmR"
                     /codon_start=1
                     /transl_table=11
                     /product="chloramphenicol acetyltransferase"
                     /protein_id="UOX75812"
                     /translation="MEKKITGYTTVDISQWHRKEHFEAFQSVAQCTYNQTVQLDITAF
                     LKTVKKNKHKFYPAFIHILARLMNAHPEFRMAMKDGELVIWDSVHPCYTVFHEQTETF
                     SSLWSEYHDDFRQFLHIYSQDVACYGENLAYFPKGFIENMFFVSANPWVSFTSFDLNV
                     ANMDNFFAPVFTMGKYYTQGDKVLMPLAIQVHHAVCDGFHVGRMLNELQQYCDEWQGG
                     A"
     regulatory      6136..6230
                     /regulatory_class="terminator"
                     /note="transcription terminator from phage lambda; lambda
                     t0 terminator"
     rep_origin      6344..6889
                     /note="Plasmids containing the medium-copy-number p15A
                     origin of replication can be propagated in E. coli cells
                     that contain a second plasmid with the ColE1 origin.; p15A
                     ori"
                     /direction=RIGHT
     gene            complement(7016..8863)
                     /gene="mutL"
                     /allele="mutL-E32K"
     CDS             complement(7016..8863)
                     /gene="mutL"
                     /allele="mutL-E32K"
                     /note="Contains E32K mutation which inhibits
                     methyl-directed mismatch repair"
                     /codon_start=1
                     /transl_table=11
                     /product="DNA mismatch repair protein MutL"
                     /protein_id="UOX75813"
                     /translation="MPIQVLPPQLANQIAAGEVVERPASVVKELVKNSLDAGATRIDI
                     DIERGGAKLIRIRDNGCGIKKDELALALARHATSKIASLDDLEAIISLGFRGEALASI
                     SSVSRLTLTSRTAEQQEAWQAYAEGRDMNVTVKPAAHPVGTTLEVLDLFYNTPARRKF
                     LRTEKTEFNHIDEIIRRIALARFDVTINLSHNGKIVRQYRAVPEGGQKERRLGAICGT
                     AFLEQALAIEWQHGDLTLRGWVADPNHTTPALAEIQYCYVNGRMMRDRLINHAIRQAC
                     EDKLGADQQPAFVLYLEIDPHQVDVNVHPAKHEVRFHQSRLVHDFIYQGVLSVLQQQL
                     ETPLPLDDEPQPAPRSIPENRVAAGRNHFAEPAAREPVAPRYTPAPASGSRPAAPWPN
                     AQPGYQKQQGEVYRQLLQTPAPMQKLKAPEPQEPALAANSQSFGRVLTIVHSDCALLE
                     RDGNISLLSLPVAERWLRQAQLTPGEAPVCAQPLLIPLRLKVSAEEKSALEKAQSALA
                     ELGIDFQSDAQHVTIRAVPLPLRQQNLQILIPELIGYLAKQSVFEPGNIAQWIARNLM
                     SEHAQWSMAQAITLLADVERLCPQLVKTPPGGLLQSVDLHPAIKALKDE"
     ncRNA           complement(8943..8992)
                     /ncRNA_class="hammerhead_ribozyme"
                     /note="hammerhead ribozyme from the tobacco ringspot virus
                     satellite RNA (Khvorova et al., 2003); sTRSV HHRz"
     regulatory      complement(8996..9081)
                     /regulatory_class="promoter"
                     /note="CymRC promoter"
     protein_bind    8996..9023
                     /note="CymR-binding P2 operator sequence from the p-cmt
                     operon of Pseudomonas putida (Mullick et al., 2006); CuO"
                     /bound_moiety="CymR"
     protein_bind    9054..9081
                     /note="CymR-binding P2 operator sequence from the p-cmt
                     operon of Pseudomonas putida (Mullick et al., 2006); CuO"
                     /bound_moiety="CymR"
     regulatory      9178..9231
                     /regulatory_class="promoter"
                     /note="LacI promoter"
ORIGIN
        1 gttgaacgta atcgttttgt tgttgaagat atgtggctgg gtgtgctggt gagccgtggt
       61 ctgagccgtg atgatgccga agatattctg tggctgattt ttaacagcgt tcgtggtctg
      121 gtagttcgta gcctgtggca gaaagataaa gaacgttttg aacgtgtgcg taatagcacc
      181 ctggaaattg cacgtgaacg ttatgcaaaa ttcaaacgtt gatcactggc cgtcgtttta
      241 caacgtcgtg acggaagcgg aatatatccc taggtataaa cgcagaaagg cccacccgaa
      301 ggtgagccag tgtgactcta gtagagagcg ttcaccgaca aacaacagat aaaacgaaag
      361 gcccagtctt tcgactgagc ctttcgtttt atttgatgcc tggagatcct tactcgagtt
      421 aagcagccag agcgtagttt tcgtcgttag cagcgtcacc tcctagctga ctcaaatcaa
      481 tgcgtgtttc ataaagacca gtgatggatt gatggataag agtggcatct aaaacttctt
      541 ttgtagacgt atatcgttta cgatcaattg ttgtatcaaa atatttaaaa gcagcgggag
      601 ctccaagatt cgtcaacgta aataaatgaa taatattttc tgcttgttca cgtattggtt
      661 tgtctctatg tttgttatat gcactaagaa ctttatctaa attggcatct gctaaaataa
      721 cacgcttaga aaattcactg atttgctcaa taatctcatc taaataatgc ttatgctgct
      781 ccacaaacaa ttgtttttgt tcgttatctt ctggactacc cttcaacttt tcataatgac
      841 tagctaaata taaaaaattc acatatttgc ttggcagagc cagctcattt cctttttgta
      901 attctccggc actagccagc atccgtttac gaccgttttc taactcaaaa agactatatt
      961 taggtagttt aatgattaag tcttttttaa cttccttata tcctttagct tctaaaaagt
     1021 caatcggatt tttttcaaag gaacttcttt ccataattgt gatccctagt aactctttaa
     1081 cggattttaa cttcttcgat ttcccttttt ccaccttagc aaccactagg actgaataag
     1141 ctaccgttgg actatcaaaa ccaccatatt tttttggatc ccagtctttt ttacgagcaa
     1201 taagcttgtc cgaatttctt tttggtaaaa ttgactcctt ggagaatccg cctgtctgta
     1261 cttctgtttt cttgacaata ttgacttggg gcatggacaa tactttgcgc actgtggcaa
     1321 aatctcgccc tttatcccag acaatttctc cagtttcccc attagtttcg attagagggc
     1381 gtttgcgaat ctctccattt gcaagtgtaa tttctgtttt gaagaagttc atgatattag
     1441 agtaaaagaa atattttgcg gttgctttgc ctatttcttg ctcagactta gcaatcattt
     1501 tacgaacatc ataaacttta taatcaccat agacaaactc cgattcaagt tttggatatt
     1561 tcttaatcaa agcagttcca acgacggcat ttagatacgc atcatgggca tgatggtaat
     1621 tgttaatctc acgtacttta tagaattgga aatcttttcg gaagtcagaa actaatttag
     1681 attttaaggt aatcacttta acctctcgaa taagtttatc attttcatcg tatttagtat
     1741 tcatgcgact atccaaaatt tgtgccacat gcttagtgat ttggcgagtt tcaaccaatt
     1801 ggcgtttgat aaaaccagct ttatcaagtt cactcaaacc tccacgttca gctttcgtta
     1861 aattatcaaa cttacgttga gtgattaact tggcgtttag aagttgtctc caatagtttt
     1921 tcatcttttt gactacttct tcacttggaa cgttatccga tttaccacga tttttatcag
     1981 aacgcgttaa gaccttattg tctattgaat cgtctttaag gaaactttgt ggaacaatgt
     2041 gatcgacatc ataatcactt aaacgattaa tatctaattc ttggtccaca tacatgtctc
     2101 ttccattttg gagataatag agatagagct tttcattttg caattgagta ttttcaacag
     2161 gatgctcttt aagaatctga cttcctaatt ctttgatacc ttcttcgatt cgtttcatac
     2221 gctctcgcga atttttctgg cccttttgag ttgtctgatt ttcacgtgcc atttcaataa
     2281 cgatattttc tggcttatgc cgccccatta ctttgaccaa ttcatcaaca acttttacag
     2341 tctgtaaaat acctttttta atagcagggc taccagctaa atttgcaata tgttcatgta
     2401 aactatcgcc ttgtccagac acttgtgctt tttgaatgtc ttctttaaat gtcaaactat
     2461 catcatggat cagctgcata aaattgcgat tggcaaaacc atctgatttc aaaaaatcta
     2521 atattgtttt gccagattgc ttatccctaa taccattaat caattttcga gacaaacgtc
     2581 cccaaccagt ataacggcga cgtttaagct gtttcatcac cttatcatca aagaggtgag
     2641 catatgtttt aagtctttcc tcaatcatct ccctatcttc aaataaggtc aatgttaaaa
     2701 caatatcctc taagatatct tcattttctt cattatccaa aaaatcttta tctttaataa
     2761 tttttagcaa atcatggtag gtacctaatg aagcattaaa tctatcttca actcctgaaa
     2821 tttcaacact atcaaaacat tctatttttt tgaaataatc ttcttttaat tgcttaacgg
     2881 ttacttttcg atttgttttg aagagtaaat caacaatggc tttcttctgt tcacctgaaa
     2941 gaaatgctgg ttttcgcatt ccttcagtaa catatttgac ctttgtcaat tcgttataaa
     3001 ccgtaaaata ctcataaagc aaactatgtt ttggtagtac tttttcattt ggaagatttt
     3061 tatcaaagtt tgtcatgcgt tcaataaatg attgagctga agcaccttta tcgacaactt
     3121 cttcaaaatt ccatggggta attgtttctt cagacttccg agtcatccat gcaaaacgac
     3181 tattgccacg cgccaatgga ccaacataat aaggaattcg aaaagtcaag attttttcaa
     3241 tcttctcacg attgtctttt aaaaatggat aaaagtcttc ttgtcttctc aaaatagcat
     3301 gcagctcacc caagtgaatt tgatggggaa tagagccgtt gtcaaaggtc cgttgcttgc
     3361 gcagcaaatc ttcacgattt agtttcacca ataattcctc agtaccatcc attttttcta
     3421 aaattggttt gataaattta taaaattctt cttggctagc tcccccatca atataacctg
     3481 catatccgtt ttttgattga tcaaaaaaga tttctttata cttttctgga agttgttgtc
     3541 gaactaaagc ttttaaaaga gtcaagtctt gatgatgttc atcgtagcgt ttaatcattg
     3601 aagctgatag gggagcctta gttatttcag tatttactct taggatatct gaaagtaaaa
     3661 tagcatctga taaattctta gctgccaaaa acaaatcagc atattgatct ccaatttgcg
     3721 ccaataaatt atctaaatca tcatcgtaag tatcttttga aagctgtaat ttagcatctt
     3781 ctgccaaatc aaaatttgat ttaaaattag gggtcaaacc caatgacaaa gcaatgagat
     3841 tcccaaataa gccatttttc ttctcaccgg ggagctgagc aatgagattt tctaatcgtc
     3901 ttgatttact caatcgtgca gaaagaatcg ctttagcatc tactccactt gcgttaatag
     3961 ggttttcttc aaataattga ttgtaggttt gtaccaactg gataaatagt ttgtccacat
     4021 cactattatc aggatttaaa tctccctcaa tcaaaaaatg accacgaaac ttaatcatat
     4081 gcgctaaggc caaatagatt aagcgcaaat ccgctttatc agtagaatct accaattttt
     4141 ttcgcagatg atagatagtt ggatatttct catgataagc aacttcatct actatatttc
     4201 caaaaatagg atgacgttca tgcttcttgt cttcttccac caaaaaagac tcttcaagtc
     4261 gatgaaagaa actatcatct actttcgcca tctcatttga aaaaatctcc tgtagataac
     4321 aaatacgatt cttccgacgt gtataccttc tacgagctgt ccgtttgaga cgagtcgctt
     4381 ccgctgtctc tccactgtca aataaaagag cccctataag attttttttg atactgtggc
     4441 ggtctgtatt tcccagaacc ttgaactttt tagacggaac cttatattca tcagtgatca
     4501 ccgcccatcc gacgctattt gtgccgatat ctaagcctat tgagtatttc ttatccatag
     4561 atccgaagtc ctctttagat cttttgaatt cttttctcta tcactgatag ggagtggtaa
     4621 aataactcta tcaacgatag agtgtcaact ttatggctag ctcagtccta ggtacaatgc
     4681 tagcaattag gaattaatga tgtctagatt agataaaagt aaagtgatta acagcgcatt
     4741 agagctgctt aatgaggtcg gaatcgaagg tttaacaacc cgtaaactcg cccagaagct
     4801 aggtgtagag cagcctacat tgtattggca tgtaaaaaat aagcgggctt tgctcgacgc
     4861 cttagccatt gagatgttag ataggcacca tactcacttt tgccctttag aaggggaaag
     4921 ctggcaagat tttttacgta ataacgctaa aagttttaga tgtgctttac taagtcatcg
     4981 cgatggagca aaagtacatt taggtacacg gcctacagaa aaacagtatg aaactctcga
     5041 aaatcaatta gcctttttat gccaacaagg tttttcacta gagaatgcat tatatgcact
     5101 cagcgctgtg gggcatttta ctttaggttg cgtattggaa gatcaagagc atcaagtcgc
     5161 taaagaagaa agggaaacac ctactactga tagtatgccg ccattattac gacaagctat
     5221 cgaattattt gatcaccaag gtgcagagcc agccttctta ttcggccttg aattgatcat
     5281 atgcggatta gaaaaacaac ttaaatgtga aagtgggtct taagacgtcg atatctggcg
     5341 aaaatgagac gttgatcggc acgtaagagg ttccaacttt caccataatg aaataagatc
     5401 actaccgggc gtattttttg agttatcgag attttcagga gctaaggaag ctaaaatgga
     5461 gaaaaaaatc actggatata ccaccgttga tatatcccaa tggcatcgta aagaacattt
     5521 tgaggcattt cagtcagttg ctcaatgtac ctataaccag accgttcagc tggatattac
     5581 ggccttttta aagaccgtaa agaaaaataa gcacaagttt tatccggcct ttattcacat
     5641 tcttgcccgc ctgatgaatg ctcatccgga atttcgtatg gcaatgaaag acggtgagct
     5701 ggtgatatgg gatagtgttc acccttgtta caccgttttc catgagcaaa ctgaaacgtt
     5761 ttcatcgctc tggagtgaat accacgacga tttccggcag tttctacaca tatattcgca
     5821 agatgtggcg tgttacggtg aaaacctggc ctatttccct aaagggttta ttgagaatat
     5881 gtttttcgtc tcagccaatc cctgggtgag tttcaccagt tttgatttaa acgtggccaa
     5941 tatggacaac ttcttcgccc ccgttttcac catgggcaaa tattatacgc aaggcgacaa
     6001 ggtgctgatg ccgctggcga ttcaggttca tcatgccgtt tgtgatggct tccatgtcgg
     6061 cagaatgctt aatgaattac aacagtactg cgatgagtgg cagggcgggg cgtaatttga
     6121 tatcgagctc gcttggactc ctgttgatag atccagtaat gacctcagaa ctccatctgg
     6181 atttgttcag aacgctcggt tgccgccggg cgttttttat tggtgagaat ccaagcacta
     6241 gtaacaactt atatcgtatg gggctgactt caggtgctac atttgaagag ataaattgca
     6301 ctgaaatcta gaaatatttt atctgattaa taagatgatc ttcttgagat cgttttggtc
     6361 tgcgcgtaat ctcttgctct gaaaacgaaa aaaccgcctt gcagggcggt ttttcgaagg
     6421 ttctctgagc taccaactct ttgaaccgag gtaactggct tggaggagcg cagtcaccaa
     6481 aacttgtcct ttcagtttag ccttaaccgg cgcatgactt caagactaac tcctctaaat
     6541 caattaccag tggctgctgc cagtggtgct tttgcatgtc tttccgggtt ggactcaaga
     6601 cgatagttac cggataaggc gcagcggtcg gactgaacgg ggggttcgtg catacagtcc
     6661 agcttggagc gaactgccta cccggaactg agtgtcaggc gtggaatgag acaaacgcgg
     6721 ccataacagc ggaatgacac cggtaaaccg aaaggcagga acaggagagc gcacgaggga
     6781 gccgccaggg ggaaacgcct ggtatcttta tagtcctgtc gggtttcgcc accactgatt
     6841 tgagcgtcag atttcgtgat gcttgtcagg ggggcggagc ctatggaaaa acggctttgc
     6901 cgcggccctc tcacttccct gttaagtatc ttcctggcat cttccaggaa atctccgccc
     6961 cacgacaggt ttcccgactg gaaagcgatg cctggagatc cttaccttac tgatatcact
     7021 catctttcag ggcttttatc gccggatgta aatcaacaga ttgtaacaga ccacccggcg
     7081 gcgttttcac aagttgcgga cataaccgtt ccacgtccgc cagcagggtt atggcctgtg
     7141 ccattgacca ctgcgcatgt tcgctcatca gatttcgtgc aatccactgc gcaatattgc
     7201 caggttcgaa tacggactgc ttcgccaggt agcctatcag ttcaggaatc aagatttgta
     7261 aattttgttg gcgtaagggt aaaggcactg ccctgatggt cacatgctgt gcatctgact
     7321 ggaaatcaat acccaattcc gccagggcag actgcgcttt ttctaatgcc gatttttctt
     7381 cggcagaaac ttttagccgc aacggaatca gcagcggctg ggcgcaaacg ggcgcttcac
     7441 ccggcgtcaa ttgtgcctga cgcagccaac gttctgccac tggcaaggat aaaagtgaaa
     7501 tgttgccgtc gcgctccagc aacgcacagt cggaatggac gatagtcagt acccgaccaa
     7561 aactctgact gttcgccgca agtgcaggtt cctgcggttc cggcgctttt aatttttgca
     7621 tcggcgcggg cgtttgcaaa agctggcgat acacttcacc ttgctgtttc tggtagcctg
     7681 gctgcgcatt cggccagggg gcagccggac gactgcctga tgctggcgca ggagtgtagc
     7741 gcggagctac cggctcacga gctgccggtt ctgcaaagtg attgcgcccc gccgccacgc
     7801 ggttttccgg aatggaacgc ggtgcaggtt ggggttcatc gtccagcggt agcggcgttt
     7861 ccagttgctg ttgtagcacg ctcagcacgc cctgatagat aaaatcatgc accagacgcg
     7921 actgatggaa acgcacttcg tgtttggcgg ggtgcacgtt gacgtccacc tgatgtgggt
     7981 cgatctccag atacaacaca aatgccggtt gctgatcggc ccccagtttg tcttcgcagg
     8041 cctggcggat cgcgtgattg atcaggcgat cgcgcatcat gcgaccgttc acgtagcaat
     8101 actgaatttc tgccagtgcg ggcgtggtgt gatttggatc ggccacccag ccgcgtagcg
     8161 tgagatcgcc gtgttgccat tcaatcgcca gcgcttgttc aagaaaagcg gtgccgcaaa
     8221 tcgcgcctaa gcgccgttct ttttgcccgc cttccggcac tgcgcggtac tgacgcacaa
     8281 ttttaccgtt atgcgacagg ttgatcgtga cgtcgaaacg cgccagcgca atgcggcgga
     8341 tgatctcatc aatgtggtta aattcggttt tctcggtgcg caggaatttg cgccgcgccg
     8401 gggtgttgta gaacagatcc agcacctcca gcgtcgtccc cacaggatgc gccgccggtt
     8461 ttaccgtcac gttcatatcg cgcccttcgg cataggcctg ccaggcttcc tgctgttctg
     8521 cggtgcgtga agtgagcgtc aggcgggaaa ccgaactgat actcgccagc gcctcaccgc
     8581 gaaagcccag gctgataatg gcttcgagat cgtccagaga ggcgatttta ctggtggcat
     8641 gacgagccag cgccagcgcc agctcatctt ttttgatacc gcagccgtta tcacgaatgc
     8701 ggataagttt cgccccaccg cgttcgatat caatatcgat acgcgtcgca cctgcatcga
     8761 ggctgttttt cactagttct ttgactaccg acgcaggtcg ctcgaccacc tcacctgcgg
     8821 caatctggtt cgccagttgt ggcggtaaga cctgaattgg catcaatcgt ccttagagtt
     8881 ttgcgagtta agcagccaga gctgcgctct tcccagttct taaacaaaat tatttgtaga
     8941 ggctgtttcg tcctcacgga ctcatcagac cggaaagcac atccggtgac agctttacaa
     9001 acagaccaga ttgtctgttt gttgaatcta ttatacagaa aaattttcca taatacaaac
     9061 agaccagatt gtctgtttgt tccggtacat tgacatatgt atcccagcca acgatcgtta
     9121 agcgggagac cagaaacaaa aaaaggcccc ccgttaggga ggccttcaat aattggggcg
     9181 gcgcgccatc gaatggcgca aaacctttcg cggtatggca tgatagcgcc caatggaata
     9241 ttcatagtaa ataccaaacg gagattctta tgagcccgaa acgtcgtacc caggcagaac
     9301 gtgcaatgga aacccagggt aaactgattg cagcagcact gggtgttctg cgtgaaaaag
     9361 gttatgcagg ttttcgtatt gcagatgttc cgggtgcagc cggtgttagc cgtggtgcac
     9421 agagccatca ttttccgacc aaactggaac tgctgctggc aacctttgaa tggctgtatg
     9481 agcagattac cgaacgtagc cgtgcacgtc tggcaaaact gaaaccggaa gatgatgtta
     9541 ttcagcagat gctggatgat gcagcagaat tttttctgga tgatgatttt agcatcggcc
     9601 tggatctgat tgttgcagca gatcgtgatc cggcactgcg tgaaggtatt cagcgtacc //
