# Supplementary material for: Efficient and iterative retron-mediated in vivo recombineering in Escherichia coli
Source: Synth Biol (Oxf). 2022 May 3;7(1):ysac007. doi: 10.1093/synbio/ysac007 (PMC9165427; doi:10.1093/synbio/ysac007)
Supplement: ysac007_Supp [file ysac007_supp.zip › PlasmidSeqs.zip!/PlasmidSeqs/pTJV1Sc2-tetA.gb]

LOCUS       OK423785                6011 bp    DNA     circular SYN 15-APR-2022
DEFINITION  Cloning vector pTJV1Sc2-tetA, complete sequence.
ACCESSION   OK423785
VERSION     OK423785
KEYWORDS    .
SOURCE      Cloning vector pTJV1Sc2-tetA
  ORGANISM  Cloning vector pTJV1Sc2-tetA
            other sequences; artificial sequences; vectors.
REFERENCE   1  (bases 1 to 6011)
  AUTHORS   Ellington,A.J. and Reisch,C.R.
  TITLE     Direct Submission
  JOURNAL   Submitted (05-OCT-2021) Microbiology and Cell Science, University
            of Florida, 1355 Museum Dr, Gainesville, FL 32611, USA
COMMENT     ##Assembly-Data-START##
            Assembly Method       :: Addgene proprietary assembly program v.
                                     03-2021
            Assembly Name         :: pTJV1Sc2-tetA
            Coverage              :: 3358x
            Sequencing Technology :: Illumina
            ##Assembly-Data-END##
FEATURES             Location/Qualifiers
     source          1..6011
                     /organism="Cloning vector pTJV1Sc2-tetA"
                     /mol_type="other DNA"
                     /db_xref="taxon:2935456"
                     /note="Addgene:166982"
     ncRNA           50..5986
                     /ncRNA_class="guide_RNA"
                     /note="guide RNA scaffold for the Streptococcus pyogenes
                     CRISPR/Cas9 system; gRNA scaffold"
     protein_bind    81..99
                     /note="bacterial operator O2 for the tetR and tetA genes;
                     tet operator; tetO"
                     /bound_moiety="tetracycline repressor TetR"
     protein_bind    106..124
                     /note="bacterial operator O2 for the tetR and tetA genes;
                     tet operator; tetO"
                     /bound_moiety="tetracycline repressor TetR"
     rep_origin      690..912
                     /note="low-copy replication origin that requires the
                     Rep101 protein; pSC101 ori"
     gene            960..1910
                     /gene="rep101 encoding an A56V mutation"
     CDS             960..1910
                     /gene="rep101 encoding an A56V mutation"
                     /note="Rep101(Ts); (Armstrong et al., 1984)"
                     /codon_start=1
                     /transl_table=11
                     /product="temperature-sensitive version of the RepA
                     protein needed for replication with the pSC101 origin"
                     /protein_id="UOX75823"
                     /translation="MSELVVFKANELAISRYDLTEHETKLILCCVALLNPTIENPTRK
                     ERTVSFTYNQYVQMMNISRENAYGVLAKATRELMTRTVEIRNPLVKGFEIFQWTNYAK
                     FSSEKLELVFSEEILPYLFQLKKFIKYNLEHVKSFENKYSMRIYEWLLKELTQKKTHK
                     ANIEISLDEFKFMLMLENNYHEFKRLNQWVLKPISKDLNTYSNMKLVVDKRGRPTDTL
                     IFQVELDRQMDLVTELENNQIKMNGDKIPTTITSDSYLHNGLRKTLHDALTAKIQLTS
                     FEAKFLSDMQSKYDLNGSFSWLTQKQRTTLENILAKYGRI"
     regulatory      2053..2139
                     /regulatory_class="terminator"
                     /note="transcription terminator T1 from the E. coli rrnB
                     gene; rrnB T1 terminator; Escherichia coli rrnB"
     CDS             complement(2157..2969)
                     /note="recombinase from colinsella stercoris phage"
                     /codon_start=1
                     /transl_table=11
                     /product="CspRecT"
                     /protein_id="UOX75824"
                     /translation="MNQIVKFTDDSGLAVQVTPDDVRRYICENATEKEVGLFLQLCQT
                     QRLNPFVKDAYLVKYGGAPASMITSYQVFNRRACRDANYDGIKSGVVVLRDGDVVHKR
                     GAACYKKAGEELIGGWAEVRFKDGRETAYAEVALDDYSTGKSNWAKMPGVMIEKCAKA
                     AAWRLAFPDTFQGMYAAEEMDQAQQPEQVRAQAEQPVDLQPIRELFKPYCEHFGITPA
                     EGMTAVCGAVGAEGMHSMTEQQARRARAWMEEEMAAPAVEAEYEVVDEGEVF"
     regulatory      complement(2986..3035)
                     /regulatory_class="promoter"
                     /note="VanCC promoter"
     CDS             complement(3047..4009)
                     /codon_start=1
                     /transl_table=11
                     /product="RNA-directed DNA polymerase from retron Ec86"
                     /protein_id="UOX75825"
                     /translation="MKSAEYLNTFRLRNLGLPVMNNLHDMSKATRISVETLRLLIYTA
                     DFRYRIYTVEKKGPEKRMRTIYQPSRELKALQGWVLRNILDKLSSSPFSIGFEKHQSI
                     LNNATPHIGANFILNIDLEDFFPSLTANKVFGVFHSLGYNRLISSVLTKICCYKNLLP
                     QGAPSSPKLANLICSKLDYRIQGYAGSRGLIYTRYADDLTLSAQSMKKVVKARDFLFS
                     IIPSEGLVINSKKTCISGPRSQRKVTGLVISQEKVGIGREKYKEIRAKIHHIFCGKSS
                     EIEHVRGWLSFILSVDSKSHRRLITYISKLEKKYGKNPLNKAKT"
     ncRNA           complement(4029..4225)
                     /ncRNA_class="other"
                     /product="msr-msd RNA coding sequence"
     regulatory      complement(4234..4264)
                     /regulatory_class="promoter"
                     /note="J23101 promoter"
     regulatory      4609..4700
                     /regulatory_class="promoter"
                     /note="AmpR promoter; bla"
     gene            4701..5492
                     /gene="aadA"
     CDS             4701..5492
                     /gene="aadA"
                     /note="Confers spectinomycin and streptomycin resistance"
                     /codon_start=1
                     /transl_table=11
                     /product="Aminoglycoside (3'') (9) adenylyltransferase"
                     /protein_id="UOX75826"
                     /translation="MREAVIAEVSTQLSEVVGVIERHLEPTLLAVHLYGSAVDGGLKP
                     HSDIDLLVTVTVRLDETTRRALINDLLETSASPGESEILRAVEVTIVVHDDIIPWRYP
                     AKRELQFGEWQRNDILAGIFEPATIDIDLAILLTKAREHSVALVGPAAEELFDPVPEQ
                     DLFEALNETLTLWNSPPDWAGDERNVVLTLSRIWYSAVTGKIAPKDVAADWAMERLPA
                     QYQPVILEARQAYLGQEEDRLASRADQLEEFVHYVKGEITKVVGK"
     regulatory      5631..5677
                     /regulatory_class="terminator"
                     /note="transcription terminator T1 from the E. coli rrnB
                     gene; rrnB T1 terminator"
ORIGIN
        1 gttgataacg gactagcctt attttaactt gctatttcta gctctaaaac actaaggcat
       61 tctgctggcg gtgctcagta tctctatcac tgatagggat gtcaatctct atcactgata
      121 gggaagaatt ccagaaatca tccttagcga aagctgataa tctcatgacc aaaatccctt
      181 aacgtgagtt ttcgttccac tgagcgtcag accccgttga tgataccgct gccttactgg
      241 gtgcattagc cagtctgaat gacctgtcac gggataatcc gaagtggtca gactggaaaa
      301 tcagagggca ggaactgctg aacagcaaaa agtcagatag caccacatag cagacccgcc
      361 ataaaacgcc ctgagaagcc cgtgacgggc ttttcttgta ttatgggtag tttccttgca
      421 tgaatccata aaaggcgcct gtagtgccat ttacccccat tcactgccag agccgtgagc
      481 gcagcgaact gaatgtcacg aaaaagacag cgactcaggt gcctgatggt cggagacaaa
      541 aggaatattc agcgatttgc ccgattgcgg ccgcaaccga gcttgcgagg gtgctactta
      601 agcctttagg gttttaaggt ctgttttgta gaggagcaaa cagcgtttgc gacatccttt
      661 tgtaatactg cggaactgac taaagtagtg agttatacac agggctggga tctattcttt
      721 ttatcttttt ttattctttc tttattctat aaattataac cacttgaata taaacaaaaa
      781 aaacacacaa aggtctagcg gaatttacag agggtctagc agaatttaca agttttccag
      841 caaaggtcta gcagaattta cagataccca caactcaaag gaaaaggact agtaattatc
      901 attgactagc ccatctcaat tggtatagtg attaaaatca cctagaccaa ttgagatgta
      961 tgtctgaatt agttgttttc aaagcaaatg aactagcgat tagtcgctat gacttaacgg
     1021 agcatgaaac caagctaatt ttatgctgtg tggcactact caaccccacg attgaaaacc
     1081 ctacaaggaa agaacggacg gtatcgttca cttataacca atacgttcag atgatgaaca
     1141 tcagtaggga aaatgcttat ggtgtattag ctaaagcaac cagagagctg atgacgagaa
     1201 ctgtggaaat caggaatcct ttggttaaag gctttgagat tttccagtgg acaaactatg
     1261 ccaagttctc aagcgaaaaa ttagaattag tttttagtga agagatattg ccttatcttt
     1321 tccagttaaa aaaattcata aaatataatc tggaacatgt taagtctttt gaaaacaaat
     1381 actctatgag gatttatgag tggttattaa aagaactaac acaaaagaaa actcacaagg
     1441 caaatataga gattagcctt gatgaattta agttcatgtt aatgcttgaa aataactacc
     1501 atgagtttaa aaggcttaac caatgggttt tgaaaccaat aagtaaagat ttaaacactt
     1561 acagcaatat gaaattggtg gttgataagc gaggccgccc gactgatacg ttgattttcc
     1621 aagttgaact agatagacaa atggatctcg taaccgaact tgagaacaac cagataaaaa
     1681 tgaatggtga caaaatacca acaaccatta catcagattc ctacctacat aacggactaa
     1741 gaaaaacact acacgatgct ttaactgcaa aaattcagct caccagtttt gaggcaaaat
     1801 ttttgagtga catgcaaagt aagtatgatc tcaatggttc gttctcatgg ctcacgcaaa
     1861 aacaacgaac cacactagag aacatactgg ctaaatacgg aaggatctga ggttcttatg
     1921 gctcttgtat ctatcagtga agcatcaaga ctaacaaaca aaagtagaac aactgttcac
     1981 cgttacgcct gcagtctaga ctcgagctga taccgctcgc cgcagccgaa cgccctaggt
     2041 ctagggcggc ggatttgtcc tactcaggag agcgttcacc gacaaacaac agataaaacg
     2101 aaaggcccag tctttcgact gagcctttcg ttttatttga tgcctctagc acgcgtttaa
     2161 aacacctcgc cctcgtcaac aacctcatac tccgcttcca cagctggcgc agccatttct
     2221 tcctccatcc aagcgcgagc acggcgagct tgctgctcgg tcatagagtg catgccttca
     2281 gcgcccaccg caccacaaac agcagtcata ccctcagccg gagtgatgcc gaagtgttcg
     2341 caatatggct tgaagagttc gcggattggc tggagatcca ctggttgctc cgcctgagcg
     2401 cgcacctgtt ctggctgttg cgcttgatcc atttcctccg cagcgtacat gccctgaaaa
     2461 gtgtccggga acgcgaggcg ccaagcagca gccttcgcgc atttttcgat cataacaccc
     2521 ggcattttcg cccaattaga tttgccggtg gaatagtcgt cgagcgccac ctcagcatac
     2581 gcagtctcgc ggccatcctt aaagcgaact tccgcccaac caccgatgag ctcctcaccc
     2641 gcctttttgt agcacgcagc accacgttta tgcacaacat caccgtcacg cagaacaacc
     2701 acaccagatt tgataccatc atagttagca tcacgacacg cgcgacggtt aaaaacttga
     2761 taggaagtaa tcatagaagc tggagcaccg ccgtatttca ccaggtaagc gtctttcaca
     2821 aacggattca gacgttgagt ctgacagagt tgcagaaaga ggcccacctc tttttcagta
     2881 gcgttctcac agatataacg gcgaacatcg tctggagtaa cttgaaccgc caggccagag
     2941 tcgtcagtga acttcacgat ttggttcatt cgttacctcc ttataattgg atccaatgat
     3001 acctaggact gagctagctg tcaattggat ccaatcaacc ggatccttag gtcttcgctt
     3061 tatttaaagg gttctttcca tatttttttt ctaatttgct aatataagtt attaatctcc
     3121 tatggctttt tgaatccaca cttaaaataa atgacaacca tcccctaacg tgttctatct
     3181 cagaagactt accgcaaaat atatgatgta tctttgctct aatttcttta tatttttctc
     3241 tacctatccc aactttctct tgtgaaataa ctaaacctgt aactttcctc tgactacgag
     3301 gcccactaat acaagttttt tttgagttaa taaccaatcc ttcacttggg attatagaaa
     3361 ataaaaaatc acgtgcttta acaacctttt tcatagactg tgcagataag gtgaggtcat
     3421 cggcatatct cgtatatatc aagccccgac tacctgcata accctgaata cgataatcaa
     3481 gtttagaaca tattagatta gctaatttag gtgatgatgg agcaccttgt ggtagcagat
     3541 ttttataaca acatattttt gtcaaaactg aagatattag tcgattataa ccaagagaat
     3601 ggaacactcc aaaaactttg ttagcagtta aacttgggaa aaaatcctcc aaatcaatat
     3661 tcagtataaa gtttgcccca atatgcgggg tagcattatt caaaatagat tggtgctttt
     3721 caaatccaat agaaaaagga gatgacgaca gtttatctaa aatgttacgt agaacccatc
     3781 cttgtaaggc tttaagttct cgagaaggtt ggtaaatggt tctcattctc ttctctgggc
     3841 ctttcttttc tacagtgtag atcctatagc gaaaatcagc tgtatagatt aacaaccgaa
     3901 gtgtttcaac agatatgcga gtcgccttag acatgtcatg caaattgttc atgacaggta
     3961 ggccgagatt tctcaatcta aaagtgttca aatattcagc ggatttcatg aaagttgcgc
     4021 acccttacgt cagaaaaaac gggtttcctg caaattgcac caacgcatac agcgccagca
     4081 gaatgccata gtgggcggtg acgtcgttcg agtgaaccag atcgcgcagg caggaaaaca
     4141 gacagtaact cagattcaat gcaggatgcc gaaacaacat ccagaggttg accttaatga
     4201 taaacctctc gctaagggtg cgcatttaat taagcaccat acctaggact gagctagctg
     4261 tcaactctag aagcggccgc gaattccaga aatcatcctt agcgaaagct aaggattttt
     4321 tttatctgaa attctgcctc gtgatacgcc tatgacgaaa gggcctcgtg atacgcctat
     4381 gacgaaaggg cctcgtgata cgcctatttt tataggttaa tgtcatgata ataatggttt
     4441 cttagacgtc gatatctggc gaaaatgaga cgttgatcgg cacgtaagca ggtggcactt
     4501 ttcggggaaa tgtgcgctga acccctattt gtttattttt ctaaatacat tcaaatatgt
     4561 atccgctcat gagacaataa ccctggtagc gaccgagtga gctagctatt tgtttatttt
     4621 tctaaataca ttcaaatatg tatccgctca tgagacaata accctgataa atgcttcaat
     4681 aatattgaaa aaggaagagt atgagggaag cggtgatcgc cgaagtatcg actcaactat
     4741 cagaggtagt tggcgtcatc gagcgccatc tcgaaccgac gttgctggcc gtacatttgt
     4801 acggctccgc agtggatggc ggcctgaagc cacacagtga tattgatttg ctggttacgg
     4861 tgaccgtaag gcttgatgaa acaacgcggc gagctttgat caacgacctt ttggaaactt
     4921 cggcttcccc tggagagagc gagattctcc gcgctgtaga agtcaccatt gttgtgcacg
     4981 acgacatcat tccgtggcgt tatccagcta agcgcgaact gcaatttgga gaatggcagc
     5041 gcaatgacat tcttgcaggt atcttcgagc cagccacgat cgacattgat ctggctatct
     5101 tgctgacaaa agcaagagaa catagcgttg ccttggtagg tccagcggcg gaggaactct
     5161 ttgatccggt tcctgaacag gatctatttg aggcgctaaa tgaaacctta acgctatgga
     5221 actcgccgcc cgactgggct ggcgatgagc gaaatgtagt gcttacgttg tcccgcattt
     5281 ggtacagcgc agtaaccggc aaaatcgcgc cgaaggatgt cgctgccgac tgggcaatgg
     5341 agcgcctgcc ggcccagtat cagcccgtca tacttgaagc tagacaggct tatcttggac
     5401 aagaagaaga tcgcttggcc tcgcgcgcag atcagttgga agaatttgtc cactacgtga
     5461 aaggcgagat caccaaggta gtcggcaaat aatgtcctgt cagcagacca agtttactca
     5521 tatatacttt agattgattt aaaacttcat ttttaattta aaaggatcta ggtgaagatc
     5581 cgcctgcagt ctagactcga gtaaggatcc agttcaccga caaacaacag ataaaacgaa
     5641 aggcccagtc tttcgactga gcctttcgtt ttatttgatg cctggcagtt ccctactctc
     5701 gcatggggag accccacact accatcggcg ctacggcgtt tcacttctga gttcggcatg
     5761 gggtcaggtg ggaccaccgc gctactgccg ccaggcaaat tctgttttat cagaccgctt
     5821 ctgcgttctg atttaatctg tatcaggctg aaaatcttct ctcatccgcc aaaacagcca
     5881 agctggagac cgtttaaact caatgatgat gatgatgatg gtcgacggcg ctattcagat
     5941 cctcttctga gatgagtttt tgttcgggcc caagcttcaa aaaaagcacc gactcggtgc
     6001 cactttttca a
//
