# Supplementary material for: Efficient and iterative retron-mediated in vivo recombineering in Escherichia coli
Source: Synth Biol (Oxf). 2022 May 3;7(1):ysac007. doi: 10.1093/synbio/ysac007 (PMC9165427; doi:10.1093/synbio/ysac007)
Supplement: ysac007_Supp [file ysac007_supp.zip › PlasmidSeqs.zip!/PlasmidSeqs/pTJV1Sc3-rpoB.gb]

LOCUS       OK483345                6138 bp    DNA     circular SYN 15-APR-2022
DEFINITION  Cloning vector pTJV1Sc3-rpoB plasmid pTJV1Sc3-rpoB, complete
            sequence.
ACCESSION   OK483345
VERSION     OK483345
KEYWORDS    .
SOURCE      Cloning vector pTJV1Sc3-rpoB
  ORGANISM  Cloning vector pTJV1Sc3-rpoB
            other sequences; artificial sequences; vectors.
REFERENCE   1  (bases 1 to 6138)
  AUTHORS   Ellington,A.J. and Reisch,C.R.
  TITLE     Direct Submission
  JOURNAL   Submitted (11-OCT-2021) Microbiology and Cell Science, University
            of Florida, 1355 Museum Dr, Gainesville, FL 32611, United States
COMMENT     ##Assembly-Data-START##
            Assembly Method       :: Addgene proprietary assembly program v.
                                     03-2021
            Assembly Name         :: pTJV1Sc3-rpoB
            Coverage              :: 38x
            Sequencing Technology :: Illumina
            ##Assembly-Data-END##
FEATURES             Location/Qualifiers
     source          1..6138
                     /organism="Cloning vector pTJV1Sc3-rpoB"
                     /mol_type="other DNA"
                     /db_xref="taxon:2935458"
                     /plasmid="pTJV1Sc3-rpoB"
                     /note="Addgene:166983"
     rep_origin      45..267
                     /note="low-copy replication origin that requires the
                     Rep101 protein; pSC101 ori"
     protein_bind    833..851
                     /note="bacterial operator O2 for the tetR and tetA genes;
                     tet operator; tetO"
                     /bound_moiety="tetracycline repressor TetR"
     protein_bind    858..876
                     /note="bacterial operator O2 for the tetR and tetA genes;
                     tet operator; tetO"
                     /bound_moiety="tetracycline repressor TetR"
     ncRNA           907..982
                     /ncRNA_class="guide_RNA"
                     /note="guide RNA scaffold for the Streptococcus pyogenes
                     CRISPR/Cas9 system; gRNA scaffold"
     regulatory      1291..1337
                     /regulatory_class="terminator"
                     /note="transcription terminator T1 from the E. coli rrnB
                     gene; rrnB T1 terminator"
     gene            complement(1476..2267)
                     /gene="aadA"
     CDS             complement(1476..2267)
                     /gene="aadA"
                     /note="confers resistance to spectinomycin and
                     streptomycin; SmR; Murphy, 1985"
                     /codon_start=1
                     /transl_table=11
                     /product="aminoglycoside adenylyltransferase"
                     /protein_id="UOX75815"
                     /translation="MREAVIAEVSTQLSEVVGVIERHLEPTLLAVHLYGSAVDGGLKP
                     HSDIDLLVTVTVRLDETTRRALINDLLETSASPGESEILRAVEVTIVVHDDIIPWRYP
                     AKRELQFGEWQRNDILAGIFEPATIDIDLAILLTKAREHSVALVGPAAEELFDPVPEQ
                     DLFEALNETLTLWNSPPDWAGDERNVVLTLSRIWYSAVTGKIAPKDVAADWAMERLPA
                     QYQPVILEARQAYLGQEEDRLASRADQLEEFVHYVKGEITKVVGK"
     regulatory      complement(2268..2359)
                     /regulatory_class="promoter"
     regulatory      2636..2679
                     /regulatory_class="terminator"
                     /note="putative bacterial transcription terminator;
                     bacterial terminator"
     regulatory      2704..2734
                     /regulatory_class="promoter"
                     /note="J23101 promoter"
     ncRNA           2743..2939
                     /ncRNA_class="other"
                     /product="msr-msd RNA"
                     /note="msr-msd RNA serves as template for reverse
                     transcription"
     CDS             2959..3921
                     /codon_start=1
                     /transl_table=11
                     /product="RNA-directed DNA polymerase from retron EC86"
                     /protein_id="UOX75817"
                     /translation="MKSAEYLNTFRLRNLGLPVMNNLHDMSKATRISVETLRLLIYTA
                     DFRYRIYTVEKKGPEKRMRTIYQPSRELKALQGWVLRNILDKLSSSPFSIGFEKHQSI
                     LNNATPHIGANFILNIDLEDFFPSLTANKVFGVFHSLGYNRLISSVLTKICCYKNLLP
                     QGAPSSPKLANLICSKLDYRIQGYAGSRGLIYTRYADDLTLSAQSMKKVVKARDFLFS
                     IIPSEGLVINSKKTCISGPRSQRKVTGLVISQEKVGIGREKYKEIRAKIHHIFCGKSS
                     EIEHVRGWLSFILSVDSKSHRRLITYISKLEKKYGKNPLNKAKT"
     regulatory      3933..3982
                     /regulatory_class="promoter"
                     /note="VanCC promoter"
     ncRNA           3985..4034
                     /ncRNA_class="hammerhead_ribozyme"
                     /note="hammerhead ribozyme from the tobacco ringspot virus
                     satellite RNA (Khvorova et al., 2003); sTRSV HHRz"
     regulatory      4079..4090
                     /regulatory_class="ribosome_binding_site"
                     /note="strong bacterial ribosome binding site (Elowitz and
                     Leibler, 2000)"
     gene            <4100..>4906
                     /gene="recT"
     CDS             <4100..4906
                     /gene="recT"
                     /codon_start=1
                     /transl_table=11
                     /product="RecT"
                     /protein_id="UOX75818"
                     /translation="TKQPPIAKADLQKTQGNRAPAAVKNSDVISFINQPSMKEQLAAA
                     LPRHMTAERMIRIATTEIRKVPALGNCDTMSFVSAIVQCSQLGLEPGSALGHAYLLPF
                     GNKNEKSGKKNVQLIIGYRGMIDLARRSGQIASLSARVVREGDEFSFEFGLDEKLIHR
                     PGENEDAPVTHVYAVARLKDGGTQFEVMTRKQIELVRSLSKAGNNGPWVTHWEEMAKK
                     TAIRRLFKYLPVSIEIQRAVSMDEKEPLTIDPADSSVLTGEYSVIDNSEE"
     regulatory      4956..5042
                     /regulatory_class="terminator"
                     /note="transcription terminator T1 from the E. coli rrnB
                     gene; rrnB T1 terminator; Escherichia coli rrnB"
     gene            complement(5185..6135)
                     /gene="rep101 encoding an A56V mutation"
     CDS             complement(5185..6135)
                     /gene="rep101 encoding an A56V mutation"
                     /note="Rep101(Ts); (Armstrong et al., 1984)"
                     /codon_start=1
                     /transl_table=11
                     /product="temperature-sensitive version of the RepA
                     protein needed for replication with the pSC101 origin"
                     /protein_id="UOX75816"
                     /translation="MSELVVFKANELAISRYDLTEHETKLILCCVALLNPTIENPTRK
                     ERTVSFTYNQYVQMMNISRENAYGVLAKATRELMTRTVEIRNPLVKGFEIFQWTNYAK
                     FSSEKLELVFSEEILPYLFQLKKFIKYNLEHVKSFENKYSMRIYEWLLKELTQKKTHK
                     ANIEISLDEFKFMLMLENNYHEFKRLNQWVLKPISKDLNTYSNMKLVVDKRGRPTDTL
                     IFQVELDRQMDLVTELENNQIKMNGDKIPTTITSDSYLHNGLRKTLHDALTAKIQLTS
                     FEAKFLSDMQSKYDLNGSFSWLTQKQRTTLENILAKYGRI"
ORIGIN
        1 tctcaattgg tctaggtgat tttaatcact ataccaattg agatgggcta gtcaatgata
       61 attactagtc cttttccttt gagttgtggg tatctgtaaa ttctgctaga cctttgctgg
      121 aaaacttgta aattctgcta gaccctctgt aaattccgct agacctttgt gtgttttttt
      181 tgtttatatt caagtggtta taatttatag aataaagaaa gaataaaaaa agataaaaag
      241 aatagatccc agccctgtgt ataactcact actttagtca gttccgcagt attacaaaag
      301 gatgtcgcaa acgctgtttg ctcctctaca aaacagacct taaaacccta aaggcttaag
      361 tagcaccctc gcaagctcgg ttgcggccgc aatcgggcaa atcgctgaat attccttttg
      421 tctccgacca tcaggcacct gagtcgctgt ctttttcgtg acattcagtt cgctgcgctc
      481 acggctctgg cagtgaatgg gggtaaatgg cactacaggc gccttttatg gattcatgca
      541 aggaaactac ccataataca agaaaagccc gtcacgggct tctcagggcg ttttatggcg
      601 ggtctgctat gtggtgctat ctgacttttt gctgttcagc agttcctgcc ctctgatttt
      661 ccagtctgac cacttcggat tatcccgtga caggtcattc agactggcta atgcacccag
      721 taaggcagcg gtatcatcaa cggggtctga cgctcagtgg aacgaaaact cacgttaagg
      781 gattttggtc atgagattat cagctttcgc taaggatgat ttctggaatt cttccctatc
      841 agtgatagag attgacatcc ctatcagtga tagagatact gagcactgtc caatcgaaac
      901 ccctgagttt tagagctaga aatagcaagt taaaataagg ctagtccgtt atcaacttga
      961 aaaagtggca ccgagtcggt gctttttttg aagcttgggc ccgaacaaaa actcatctca
     1021 gaagaggatc tgaatagcgc cgtcgaccat catcatcatc atcattgagt ttaaacggtc
     1081 tccagcttgg ctgttttggc ggatgagaga agattttcag cctgatacag attaaatcag
     1141 aacgcagaag cggtctgata aaacagaatt tgcctggcgg cagtagcgcg gtggtcccac
     1201 ctgaccccat gccgaactca gaagtgaaac gccgtagcgc cgatggtagt gtggggtctc
     1261 cccatgcgag agtagggaac tgccaggcat caaataaaac gaaaggctca gtcgaaagac
     1321 tgggcctttc gttttatctg ttgtttgtcg gtgaactgga tccttactcg agtctagact
     1381 gcaggcggat cttcacctag atccttttaa attaaaaatg aagttttaaa tcaatctaaa
     1441 gtatatatga gtaaacttgg tctgctgaca ggacattatt tgccgactac cttggtgatc
     1501 tcgcctttca cgtagtggac aaattcttcc aactgatctg cgcgcgaggc caagcgatct
     1561 tcttcttgtc caagataagc ctgtctagct tcaagtatga cgggctgata ctgggccggc
     1621 aggcgctcca ttgcccagtc ggcagcgaca tccttcggcg cgattttgcc ggttactgcg
     1681 ctgtaccaaa tgcgggacaa cgtaagcact acatttcgct catcgccagc ccagtcgggc
     1741 ggcgagttcc atagcgttaa ggtttcattt agcgcctcaa atagatcctg ttcaggaacc
     1801 ggatcaaaga gttcctccgc cgctggacct accaaggcaa cgctatgttc tcttgctttt
     1861 gtcagcaaga tagccagatc aatgtcgatc gtggctggct cgaagatacc tgcaagaatg
     1921 tcattgcgct gccattctcc aaattgcagt tcgcgcttag ctggataacg ccacggaatg
     1981 atgtcgtcgt gcacaacaat ggtgacttct acagcgcgga gaatctcgct ctctccaggg
     2041 gaagccgaag tttccaaaag gtcgttgatc aaagctcgcc gcgttgtttc atcaagcctt
     2101 acggtcaccg taaccagcaa atcaatatca ctgtgtggct tcaggccgcc atccactgcg
     2161 gagccgtaca aatgtacggc cagcaacgtc ggttcgagat ggcgctcgat gacgccaact
     2221 acctctgata gttgagtcga tacttcggcg atcaccgctt ccctcatact cttccttttt
     2281 caatattatt gaagcattta tcagggttat tgtctcatga gcggatacat atttgaatgt
     2341 atttagaaaa ataaacaaat agctagctca ctcggtcgct accagggtta ttgtctcatg
     2401 agcggataca tatttgaatg tatttagaaa aataaacaaa taggggttca gcgcacattt
     2461 ccccgaaaag tgccacctgc ttacgtgccg atcaacgtct cattttcgcc agatatcgac
     2521 gtctaagaaa ccattattat catgacatta acctataaaa ataggcgtat cacgaggccc
     2581 tttcgtcata ggcgtatcac gaggcccttt cgtcataggc gtatcacgag gcagaatttc
     2641 agataaaaaa aatccttagc tttcgctaag gatgatttct ggaattcgcg gccgcttcta
     2701 gagttgacag ctagctcagt cctaggtatg gtgcttaatt aaatgcgcac ccttagcgag
     2761 aggtttatca ttaaggtcaa cctctggatg ttgtttcggc atcctgcatt gaatctgagt
     2821 tactgtctgt tttcctgcac ccgactcact acggtcgcgt atgtccaatc gaaaccttgg
     2881 aaggtccgaa catcggtctg atcaactctc tgtccgtcag gaaacccgtt ttttctgacg
     2941 taagggtgcg caactttcat gaaatccgct gaatatttga acacttttag attgagaaat
     3001 ctcggcctac ctgtcatgaa caatttgcat gacatgtcta aggcgactcg catatctgtt
     3061 gaaacacttc ggttgttaat ctatacagct gattttcgct ataggatcta cactgtagaa
     3121 aagaaaggcc cagagaagag aatgagaacc atttaccaac cttctcgaga acttaaagcc
     3181 ttacaaggat gggttctacg taacatttta gataaactgt cgtcatctcc tttttctatt
     3241 ggatttgaaa agcaccaatc tattttgaat aatgctaccc cgcatattgg ggcaaacttt
     3301 atactgaata ttgatttgga ggattttttc ccaagtttaa ctgctaacaa agtttttgga
     3361 gtgttccatt ctcttggtta taatcgacta atatcttcag ttttgacaaa aatatgttgt
     3421 tataaaaatc tgctaccaca aggtgctcca tcatcaccta aattagctaa tctaatatgt
     3481 tctaaacttg attatcgtat tcagggttat gcaggtagtc ggggcttgat atatacgaga
     3541 tatgccgatg acctcacctt atctgcacag tctatgaaaa aggttgttaa agcacgtgat
     3601 tttttatttt ctataatccc aagtgaagga ttggttatta actcaaaaaa aacttgtatt
     3661 agtgggcctc gtagtcagag gaaagttaca ggtttagtta tttcacaaga gaaagttggg
     3721 ataggtagag aaaaatataa agaaattaga gcaaagatac atcatatatt ttgcggtaag
     3781 tcttctgaga tagaacacgt taggggatgg ttgtcattta ttttaagtgt ggattcaaaa
     3841 agccatagga gattaataac ttatattagc aaattagaaa aaaaatatgg aaagaaccct
     3901 ttaaataaag cgaagaccta aggatccggt tgattggatc caattgacag ctagctcagt
     3961 cctaggtacc attggatcca atagctgtca ccggatgtgc tttccggtct gatgagtccg
     4021 tgaggacgaa acagcctcta caaataattt tgtttaagaa ctgggaagag cgcagattaa
     4081 agaggagaaa tactagatga ctaagcaacc accaatcgca aaagccgatc tgcaaaaaac
     4141 tcagggaaac cgtgcaccag cagcagttaa aaatagcgac gtgattagtt ttattaacca
     4201 gccatcaatg aaagagcaac tggcagcagc tcttccacgc catatgacgg ctgaacgtat
     4261 gatccgtatc gccaccacag aaattcgtaa agttccggcg ttaggaaact gtgacactat
     4321 gagttttgtc agtgcgatcg tacagtgttc acagctcgga cttgagccag gtagcgccct
     4381 cggtcatgca tatttactgc cttttggtaa taaaaacgaa aagagcggta aaaagaacgt
     4441 tcagctaatc attggctatc gcggcatgat tgatctggct cgccgttctg gtcaaatcgc
     4501 cagcctgtca gcccgtgttg tccgtgaagg tgacgagttt agcttcgaat ttggccttga
     4561 tgaaaagtta atacaccgcc cgggagaaaa cgaagatgcc ccggttaccc acgtctatgc
     4621 tgtcgcaaga ctgaaagacg gaggtactca gtttgaagtt atgacgcgca aacagattga
     4681 gctggtgcgc agcctgagta aagctggtaa taacgggccg tgggtaactc actgggaaga
     4741 aatggcaaag aaaacggcta ttcgtcgcct gttcaaatat ctgcccgtat caattgagat
     4801 ccagcgtgca gtatcaatgg atgaaaagga accactgaca atcgatcctg cagattcctc
     4861 tgtattaacc ggggaataca gtgtaatcga taattcagag gaataattca gcctggcggt
     4921 gtaatggctt tccagtcggg aaacgctaga ggcatcaaat aaaacgaaag gctcagtcga
     4981 aagactgggc ctttcgtttt atctgttgtt tgtcggtgaa cgctctcctg agtaggacaa
     5041 atccgccgcc ctagacctag ggcgttcggc tgcggcgagc ggtatcagct cgagtctaga
     5101 ctgcaggcgt aacggtgaac agttgttcta cttttgtttg ttagtcttga tgcttcactg
     5161 atagatacaa gagccataag aacctcagat ccttccgtat ttagccagta tgttctctag
     5221 tgtggttcgt tgtttttgcg tgagccatga gaacgaacca ttgagatcat acttactttg
     5281 catgtcactc aaaaattttg cctcaaaact ggtgagctga atttttgcag ttaaagcatc
     5341 gtgtagtgtt tttcttagtc cgttatgtag gtaggaatct gatgtaatgg ttgttggtat
     5401 tttgtcacca ttcattttta tctggttgtt ctcaagttcg gttacgagat ccatttgtct
     5461 atctagttca acttggaaaa tcaacgtatc agtcgggcgg cctcgcttat caaccaccaa
     5521 tttcatattg ctgtaagtgt ttaaatcttt acttattggt ttcaaaaccc attggttaag
     5581 ccttttaaac tcatggtagt tattttcaag cattaacatg aacttaaatt catcaaggct
     5641 aatctctata tttgccttgt gagttttctt ttgtgttagt tcttttaata accactcata
     5701 aatcctcata gagtatttgt tttcaaaaga cttaacatgt tccagattat attttatgaa
     5761 tttttttaac tggaaaagat aaggcaatat ctcttcacta aaaactaatt ctaatttttc
     5821 gcttgagaac ttggcatagt ttgtccactg gaaaatctca aagcctttaa ccaaaggatt
     5881 cctgatttcc acagttctcg tcatcagctc tctggttgct ttagctaata caccataagc
     5941 attttcccta ctgatgttca tcatctgaac gtattggtta taagtgaacg ataccgtccg
     6001 ttctttcctt gtagggtttt caatcgtggg gttgagtagt gccacacagc ataaaattag
     6061 cttggtttca tgctccgtta agtcatagcg actaatcgct agttcatttg ctttgaaaac
     6121 aactaattca gacataca
//
