# Supplementary material for: Efficient and iterative retron-mediated in vivo recombineering in Escherichia coli
Source: Synth Biol (Oxf). 2022 May 3;7(1):ysac007. doi: 10.1093/synbio/ysac007 (PMC9165427; doi:10.1093/synbio/ysac007)
Supplement: ysac007_Supp [file ysac007_supp.zip › PlasmidSeqs.zip!/PlasmidSeqs/pTJV1Sc-rpoB.gb]

LOCUS       OK377063                5984 bp    DNA     circular SYN 15-APR-2022
DEFINITION  Cloning vector pTJV1Sc-rpoB, complete sequence.
ACCESSION   OK377063
VERSION     OK377063
KEYWORDS    .
SOURCE      Cloning vector pTJV1Sc-rpoB
  ORGANISM  Cloning vector pTJV1Sc-rpoB
            other sequences; artificial sequences; vectors.
REFERENCE   1  (bases 1 to 5984)
  AUTHORS   Ellington,A.J. and Reisch,C.R.
  TITLE     Direct Submission
  JOURNAL   Submitted (04-OCT-2021) Microbiology and Cell Science, University
            of Florida, 1355 Museum Dr, Gainesville, FL 32611, USA
COMMENT     ##Assembly-Data-START##
            Assembly Method       :: Addgene proprietary assembly program v.
                                     03-2021
            Assembly Name         :: pTJV1Sc-rpoB
            Coverage              :: 450x
            Sequencing Technology :: Illumina
            ##Assembly-Data-END##
FEATURES             Location/Qualifiers
     source          1..5984
                     /organism="Cloning vector pTJV1Sc-rpoB"
                     /mol_type="other DNA"
                     /db_xref="taxon:2935455"
                     /note="Addgene:166978"
     regulatory      233..251
                     /regulatory_class="transcriptional_cis_regulatory_region"
                     /note="tet operator site"
     regulatory      258..276
                     /regulatory_class="transcriptional_cis_regulatory_region"
                     /note="tet operator site"
     ncRNA           307..382
                     /ncRNA_class="other"
                     /product="gRNA scaffold"
     regulatory      691..737
                     /regulatory_class="terminator"
                     /note="rrnB T1 terminator"
     gene            complement(876..1667)
                     /gene="aadA"
     CDS             complement(876..1667)
                     /gene="aadA"
                     /EC_number="2.7.7.47"
                     /note="Confers resistance to spectinomycin and
                     streptomycin"
                     /codon_start=1
                     /transl_table=11
                     /product="Aminoglycoside (3'') (9) adenylyltransferase"
                     /protein_id="UOX75819"
                     /translation="MREAVIAEVSTQLSEVVGVIERHLEPTLLAVHLYGSAVDGGLKP
                     HSDIDLLVTVTVRLDETTRRALINDLLETSASPGESEILRAVEVTIVVHDDIIPWRYP
                     AKRELQFGEWQRNDILAGIFEPATIDIDLAILLTKAREHSVALVGPAAEELFDPVPEQ
                     DLFEALNETLTLWNSPPDWAGDERNVVLTLSRIWYSAVTGKIAPKDVAADWAMERLPA
                     QYQPVILEARQAYLGQEEDRLASRADQLEEFVHYVKGEITKVVGK"
     regulatory      complement(1668..1759)
                     /regulatory_class="promoter"
                     /note="AmpR promoter"
     regulatory      2104..2134
                     /regulatory_class="promoter"
                     /note="J23101 promoter"
     ncRNA           2143..2339
                     /ncRNA_class="other"
                     /product="msr-msd sequence"
     CDS             2359..3321
                     /EC_number="2.7.7.49"
                     /note="Generates ssDNA using the msr-msd RNA as template"
                     /codon_start=1
                     /transl_table=11
                     /product="RNA-directed DNA polymerase from retron Ec86"
                     /protein_id="UOX75820"
                     /translation="MKSAEYLNTFRLRNLGLPVMNNLHDMSKATRISVETLRLLIYTA
                     DFRYRIYTVEKKGPEKRMRTIYQPSRELKALQGWVLRNILDKLSSSPFSIGFEKHQSI
                     LNNATPHIGANFILNIDLEDFFPSLTANKVFGVFHSLGYNRLISSVLTKICCYKNLLP
                     QGAPSSPKLANLICSKLDYRIQGYAGSRGLIYTRYADDLTLSAQSMKKVVKARDFLFS
                     IIPSEGLVINSKKTCISGPRSQRKVTGLVISQEKVGIGREKYKEIRAKIHHIFCGKSS
                     EIEHVRGWLSFILSVDSKSHRRLITYISKLEKKYGKNPLNKAKT"
     regulatory      3333..3382
                     /regulatory_class="promoter"
                     /note="VanCC promoter"
     gene            3399..4184
                     /gene="lambda bet"
     CDS             3399..4184
                     /gene="lambda bet"
                     /note="mediates pairing of ssDNA with complimentary
                     target"
                     /codon_start=1
                     /transl_table=11
                     /product="Beta Recombinase"
                     /protein_id="UOX75821"
                     /translation="MSTALATLAGKLAERVGMDSVDPQELITTLRQTAFKGDASDAQF
                     IALLIVANQYGLNPWTKEIYAFPDKQNGIVPVVGVDGWSRIINENQQFDGMDFEQDNE
                     SCTCRIYRKDRNHPICVTEWMDECRREPFKTREGREITGPWQSHPKRMLRHKAMIQCA
                     RLAFGFAGIYDKDEAERIVENTAYTAERQPERDITPVNDETMQEINTLLIALDKTWDD
                     DLLPLCSQIFRRDIRASSELTQAEAVKALGFLKQKAAEQKVAA"
     regulatory      4202..4288
                     /regulatory_class="terminator"
                     /note="rrnB T1 terminator"
     gene            complement(4431..5381)
                     /gene="repA101-ts"
                     /note="Encodes temperature sensitive replication
                     initiation protein"
     CDS             complement(4431..5381)
                     /gene="repA101-ts"
                     /codon_start=1
                     /transl_table=11
                     /product="RepA101-ts"
                     /protein_id="UOX75822"
                     /translation="MSELVVFKANELAISRYDLTEHETKLILCCVALLNPTIENPTRK
                     ERTVSFTYNQYVQMMNISRENAYGVLAKATRELMTRTVEIRNPLVKGFEIFQWTNYAK
                     FSSEKLELVFSEEILPYLFQLKKFIKYNLEHVKSFENKYSMRIYEWLLKELTQKKTHK
                     ANIEISLDEFKFMLMLENNYHEFKRLNQWVLKPISKDLNTYSNMKLVVDKRGRPTDTL
                     IFQVELDRQMDLVTELENNQIKMNGDKIPTTITSDSYLHNGLRKTLHDALTAKIQLTS
                     FEAKFLSDMQSKYDLNGSFSWLTQKQRTTLENILAKYGRI"
     regulatory      5429..5651
                     /regulatory_class="replication_regulatory_region"
                     /note="oriR101 origin of replication"
ORIGIN
        1 ggtctgctat gtggtgctat ctgacttttt gctgttcagc agttcctgcc ctctgatttt
       61 ccagtctgac cacttcggat tatcccgtga caggtcattc agactggcta atgcacccag
      121 taaggcagcg gtatcatcaa cggggtctga cgctcagtgg aacgaaaact cacgttaagg
      181 gattttggtc atgagattat cagctttcgc taaggatgat ttctggaatt cttccctatc
      241 agtgatagag attgacatcc ctatcagtga tagagatact gagcactgtc caatcgaaac
      301 ccctgagttt tagagctaga aatagcaagt taaaataagg ctagtccgtt atcaacttga
      361 aaaagtggca ccgagtcggt gctttttttg aagcttgggc ccgaacaaaa actcatctca
      421 gaagaggatc tgaatagcgc cgtcgaccat catcatcatc atcattgagt ttaaacggtc
      481 tccagcttgg ctgttttggc ggatgagaga agattttcag cctgatacag attaaatcag
      541 aacgcagaag cggtctgata aaacagaatt tgcctggcgg cagtagcgcg gtggtcccac
      601 ctgaccccat gccgaactca gaagtgaaac gccgtagcgc cgatggtagt gtggggtctc
      661 cccatgcgag agtagggaac tgccaggcat caaataaaac gaaaggctca gtcgaaagac
      721 tgggcctttc gttttatctg ttgtttgtcg gtgaactgga tccttactcg agtctagact
      781 gcaggcggat cttcacctag atccttttaa attaaaaatg aagttttaaa tcaatctaaa
      841 gtatatatga gtaaacttgg tctgctgaca ggacattatt tgccgactac cttggtgatc
      901 tcgcctttca cgtagtggac aaattcttcc aactgatctg cgcgcgaggc caagcgatct
      961 tcttcttgtc caagataagc ctgtctagct tcaagtatga cgggctgata ctgggccggc
     1021 aggcgctcca ttgcccagtc ggcagcgaca tccttcggcg cgattttgcc ggttactgcg
     1081 ctgtaccaaa tgcgggacaa cgtaagcact acatttcgct catcgccagc ccagtcgggc
     1141 ggcgagttcc atagcgttaa ggtttcattt agcgcctcaa atagatcctg ttcaggaacc
     1201 ggatcaaaga gttcctccgc cgctggacct accaaggcaa cgctatgttc tcttgctttt
     1261 gtcagcaaga tagccagatc aatgtcgatc gtggctggct cgaagatacc tgcaagaatg
     1321 tcattgcgct gccattctcc aaattgcagt tcgcgcttag ctggataacg ccacggaatg
     1381 atgtcgtcgt gcacaacaat ggtgacttct acagcgcgga gaatctcgct ctctccaggg
     1441 gaagccgaag tttccaaaag gtcgttgatc aaagctcgcc gcgttgtttc atcaagcctt
     1501 acggtcaccg taaccagcaa atcaatatca ctgtgtggct tcaggccgcc atccactgcg
     1561 gagccgtaca aatgtacggc cagcaacgtc ggttcgagat ggcgctcgat gacgccaact
     1621 acctctgata gttgagtcga tacttcggcg atcaccgctt ccctcatact cttccttttt
     1681 caatattatt gaagcattta tcagggttat tgtctcatga gcggatacat atttgaatgt
     1741 atttagaaaa ataaacaaat agctagctca ctcggtcgct accagggtta ttgtctcatg
     1801 agcggataca tatttgaatg tatttagaaa aataaacaaa taggggttca gcgcacattt
     1861 ccccgaaaag tgccacctgc ttacgtgccg atcaacgtct cattttcgcc agatatcgac
     1921 gtctaagaaa ccattattat catgacatta acctataaaa ataggcgtat cacgaggccc
     1981 tttcgtcata ggcgtatcac gaggcccttt cgtcataggc gtatcacgag gcagaatttc
     2041 agataaaaaa aatccttagc tttcgctaag gatgatttct ggaattcgcg gccgcttcta
     2101 gagttgacag ctagctcagt cctaggtatg gtgcttaatt aaatgcgcac ccttagcgag
     2161 aggtttatca ttaaggtcaa cctctggatg ttgtttcggc atcctgcatt gaatctgagt
     2221 tactgtctgt tttcctgcac ccgactcact acggtcgcgt atgtccaatc gaaaccttgg
     2281 aaggtccgaa catcggtctg atcaactctc tgtccgtcag gaaacccgtt ttttctgacg
     2341 taagggtgcg caactttcat gaaatccgct gaatatttga acacttttag attgagaaat
     2401 ctcggcctac ctgtcatgaa caatttgcat gacatgtcta aggcgactcg catatctgtt
     2461 gaaacacttc ggttgttaat ctatacagct gattttcgct ataggatcta cactgtagaa
     2521 aagaaaggcc cagagaagag aatgagaacc atttaccaac cttctcgaga acttaaagcc
     2581 ttacaaggat gggttctacg taacatttta gataaactgt cgtcatctcc tttttctatt
     2641 ggatttgaaa agcaccaatc tattttgaat aatgctaccc cgcatattgg ggcaaacttt
     2701 atactgaata ttgatttgga ggattttttc ccaagtttaa ctgctaacaa agtttttgga
     2761 gtgttccatt ctcttggtta taatcgacta atatcttcag ttttgacaaa aatatgttgt
     2821 tataaaaatc tgctaccaca aggtgctcca tcatcaccta aattagctaa tctaatatgt
     2881 tctaaacttg attatcgtat tcagggttat gcaggtagtc ggggcttgat atatacgaga
     2941 tatgccgatg acctcacctt atctgcacag tctatgaaaa aggttgttaa agcacgtgat
     3001 tttttatttt ctataatccc aagtgaagga ttggttatta actcaaaaaa aacttgtatt
     3061 agtgggcctc gtagtcagag gaaagttaca ggtttagtta tttcacaaga gaaagttggg
     3121 ataggtagag aaaaatataa agaaattaga gcaaagatac atcatatatt ttgcggtaag
     3181 tcttctgaga tagaacacgt taggggatgg ttgtcattta ttttaagtgt ggattcaaaa
     3241 agccatagga gattaataac ttatattagc aaattagaaa aaaaatatgg aaagaaccct
     3301 ttaaataaag cgaagaccta aggatccggt tgattggatc caattgacag ctagctcagt
     3361 cctaggtatc attggatcca attataagga ggtaacgaat gagtactgca ctcgcaacgc
     3421 tggctgggaa gctggctgaa cgtgtcggca tggattctgt cgacccacag gaactgatca
     3481 ccactcttcg ccagacggca tttaaaggtg atgccagcga tgcgcagttc atcgcattac
     3541 tgatcgttgc caaccagtac ggccttaatc cgtggacgaa agaaatttac gcctttcctg
     3601 ataagcagaa tggcatcgtt ccggtggtgg gcgttgatgg ctggtcccgc atcatcaatg
     3661 aaaaccagca gtttgatggc atggactttg agcaggacaa tgaatcctgt acatgccgga
     3721 tttaccgcaa ggaccgtaat catccgatct gcgttaccga atggatggat gaatgccgcc
     3781 gcgaaccatt caaaactcgc gaaggcagag aaatcacggg gccgtggcag tcgcatccca
     3841 aacggatgtt acgtcataaa gccatgattc agtgtgcccg tctggccttc ggatttgctg
     3901 gtatctatga caaggatgaa gccgagcgca ttgtcgaaaa tactgcatac actgcagaac
     3961 gtcagccgga acgcgacatc actccggtta acgatgaaac catgcaggag attaacactc
     4021 tgctgatcgc cctggataaa acatgggatg acgacttatt gccgctctgt tcccagatat
     4081 ttcgccgcga cattcgtgca tcgtcagaac tgacacaggc cgaagcagta aaagctcttg
     4141 gattcctgaa acagaaagcc gcagagcaga aggtggcagc atgaacgcgt gctagaggca
     4201 tcaaataaaa cgaaaggctc agtcgaaaga ctgggccttt cgttttatct gttgtttgtc
     4261 ggtgaacgct ctcctgagta ggacaaatcc gccgccctag acctagggcg ttcggctgcg
     4321 gcgagcggta tcagctcgag tctagactgc aggcgtaacg gtgaacagtt gttctacttt
     4381 tgtttgttag tcttgatgct tcactgatag atacaagagc cataagaacc tcagatcctt
     4441 ccgtatttag ccagtatgtt ctctagtgtg gttcgttgtt tttgcgtgag ccatgagaac
     4501 gaaccattga gatcatactt actttgcatg tcactcaaaa attttgcctc aaaactggtg
     4561 agctgaattt ttgcagttaa agcatcgtgt agtgtttttc ttagtccgtt atgtaggtag
     4621 gaatctgatg taatggttgt tggtattttg tcaccattca tttttatctg gttgttctca
     4681 agttcggtta cgagatccat ttgtctatct agttcaactt ggaaaatcaa cgtatcagtc
     4741 gggcggcctc gcttatcaac caccaatttc atattgctgt aagtgtttaa atctttactt
     4801 attggtttca aaacccattg gttaagcctt ttaaactcat ggtagttatt ttcaagcatt
     4861 aacatgaact taaattcatc aaggctaatc tctatatttg ccttgtgagt tttcttttgt
     4921 gttagttctt ttaataacca ctcataaatc ctcatagagt atttgttttc aaaagactta
     4981 acatgttcca gattatattt tatgaatttt tttaactgga aaagataagg caatatctct
     5041 tcactaaaaa ctaattctaa tttttcgctt gagaacttgg catagtttgt ccactggaaa
     5101 atctcaaagc ctttaaccaa aggattcctg atttccacag ttctcgtcat cagctctctg
     5161 gttgctttag ctaatacacc ataagcattt tccctactga tgttcatcat ctgaacgtat
     5221 tggttataag tgaacgatac cgtccgttct ttccttgtag ggttttcaat cgtggggttg
     5281 agtagtgcca cacagcataa aattagcttg gtttcatgct ccgttaagtc atagcgacta
     5341 atcgctagtt catttgcttt gaaaacaact aattcagaca tacatctcaa ttggtctagg
     5401 tgattttaat cactatacca attgagatgg gctagtcaat gataattact agtccttttc
     5461 ctttgagttg tgggtatctg taaattctgc tagacctttg ctggaaaact tgtaaattct
     5521 gctagaccct ctgtaaattc cgctagacct ttgtgtgttt tttttgttta tattcaagtg
     5581 gttataattt atagaataaa gaaagaataa aaaaagataa aaagaataga tcccagccct
     5641 gtgtataact cactacttta gtcagttccg cagtattaca aaaggatgtc gcaaacgctg
     5701 tttgctcctc tacaaaacag accttaaaac cctaaaggct taagtagcac cctcgcaagc
     5761 tcggttgcgg ccgcaatcgg gcaaatcgct gaatattcct tttgtctccg accatcaggc
     5821 acctgagtcg ctgtcttttt cgtgacattc agttcgctgc gctcacggct ctggcagtga
     5881 atgggggtaa atggcactac aggcgccttt tatggattca tgcaaggaaa ctacccataa
     5941 tacaagaaaa gcccgtcacg ggcttctcag ggcgttttat ggcg //
